# The Kinetochore Protein Spc105, a Novel Interaction Partner of LaeA, Regulates Development and Secondary Metabolism in *Aspergillus flavus*

**DOI:** 10.3389/fmicb.2019.01881

**Published:** 2019-08-13

**Authors:** Qing-Qing Zhi, Lei He, Jie-Ying Li, Jing Li, Zhen-Long Wang, Guang-Yao He, Zhu-Mei He

**Affiliations:** ^1^The Guangdong Province Key Laboratory for Aquatic Economic Animals, School of Life Sciences, Sun Yat-sen University, Guangzhou, China; ^2^Cancer Hospital and Institute of Guangzhou Medical University, Guangzhou, China

**Keywords:** *Aspergillus flavus*, aflatoxin, Spc105, LaeA, secondary metabolism, protein interaction

## Abstract

Nuclear protein LaeA is known as the global regulator of secondary metabolism in *Aspergillus*. LaeA connects with VeA and VelB to form a heterotrimeric complex, which coordinates fungal development and secondary metabolism. Here, we describe a new interaction partner of LaeA, the kinetochore protein Spc105, from the aflatoxin-producing fungus *Aspergillus flavus*. We showed that in addition to involvement in nuclear division, Spc105 is required for normal conidiophore development and sclerotia production of *A. flavus*. Moreover, Spc105 positively regulates the production of secondary metabolites such as aflatoxin and kojic acid, and negatively regulates the production of cyclopiazonic acid. Transcriptome analysis of the *Δspc105* strain revealed that 23 backbone genes were differentially expressed, corresponding to 19 of the predicted 56 secondary metabolite gene clusters, suggesting a broad regulatory role of Spc105 in secondary metabolism. Notably, the reduced expression of *laeA* in our transcriptome data led to the discovery of the correlation between Spc105 and LaeA, and double mutant analysis indicated a functional interdependence between Spc105 and LaeA. Further, *in vitro* and *in vivo* protein interaction assays revealed that Spc105 interacts directly with the *S*-adenosylmethionine (SAM)-binding domain of LaeA, and that the leucine zipper motif in Spc105 is required for this interaction. The Spc105-LaeA interaction identified in our study indicates a cooperative interplay of distinct regulators in *A. flavus*, providing new insights into fungal secondary metabolism regulation networks.

## Introduction

*Aspergillus flavus* is a ubiquitous saprophytic filamentous fungus that infects economically important crops such as peanuts, maize, and many other seed crops during preharvest or storage (Amaike and Keller, 2011). Infestations of *A. flavus* in crops are spread by the production and dissemination of airborne conidia or resistant structures called sclerotia. *A. flavus* can produce several types of mycotoxins known as aflatoxins (AF), aflatrem, and cyclopiazonic acid (CPA). AF is the most toxic and carcinogenic secondary metabolite, with its contamination resulting in huge economic losses and threatening human health (Abbas et al., 2017; Akhund et al., 2017). Therefore, studies on AF biosynthesis regulatory mechanisms is vital for the development of strategies to control mycotoxin contamination.

The *A. flavus* genome has been predicted to contain 56 secondary metabolite clusters, with each cluster containing “backbone” enzyme gene(s) essential to secondary metabolite biosynthesis (Georgianna et al., 2010; Marui et al., 2011). The AF cluster, containing approximately 30 genes and two pathway-specific regulatory genes (*aflR* and *aflS*), has been well characterized (Yu, 2012; Amare and Keller, 2014), and extensive research has been carried out to elucidate the genetic networks that regulate AF biosynthesis (Amare and Keller, 2014). A series of regulatory factors outside of the AF cluster have been proved to control AF biosynthesis, including the velvet complex (Bayram et al., 2008), transcription factors such as NsdC, NsdD, MeaB, and mtfA (Cary et al., 2012; Amaike et al., 2013; Zhuang et al., 2016), oxidative stress response-related genes (Linz et al., 2013; Zhao et al., 2018), and epigenetic modifications including chromatin remodeling and histone acetylation (Lan et al., 2016; Pfannenstiel et al., 2018). The involvement of these distinct regulatory factors suggests that a multilevel complex regulatory network governs AF biosynthesis.

The conserved heterotrimeric velvet complex, composed of LaeA, VeA, and VelB, is an important regulatory unit in filamentous fungi that couples light-responsive development and secondary metabolism (Bayram et al., 2008). The nuclear protein LaeA, which contains an *S*-adenosylmethionine (SAM)-binding motif required for its function, is known as a global regulator of secondary metabolism in *Aspergillus* (Bok and Keller, 2004). LaeA is required for the transcriptional activation of *aflR* as well as AF production, and transcriptional profiling of *laeA* mutant strains revealed its global regulatory role in secondary metabolite gene clusters (Kale et al., 2008; Georgianna et al., 2010). It has been suggested that LaeA controls secondary metabolism epigenetically by altering the chromatin structure to modify the gene expression (Reyes-Dominguez et al., 2010). Additionally, LaeA was also shown to control developmental differentiation, such as conidiation in various fungi (Jin et al., 2005; Chang et al., 2012; Wang et al., 2016; Lind et al., 2018). Studies in *Aspergillus nidulans* revealed that LaeA directs the formation of the LaeA-VeA-VelB velvet complex and VelB-VosA complex, the second complex that controls both sexual and asexual development (Bayram et al., 2008, 2010). In *A. flavus*, *laeA* deletion mutants displayed loss of sclerotia formation as well as reduction in conidiation, and were less capable of colonizing peanut and maize seeds (Kale et al., 2008; Chang et al., 2012).

Polarized growth is one of the distinguishing features of filamentous fungi. In *A. nidulans*, a conidium grows isotropically until first mitosis, and then switches to polarized growth and forms an elongating germ tube, a process that is tightly coordinated with nuclear division (Kang et al., 2013). A critical aspect of nuclear division is the interaction of the kinetochore with spindle microtubules. Kinetochore is a multiprotein complex assembled on the centromeres that serves as an interface between chromosomes and spindle microtubules during cell division (Guse et al., 2011). The kinetochore functions as a mechanical latch that hooks onto microtubules to support the directional movement of chromosomes and acts as a sensor of proper microtubule attachment, which is coupled with the spindle checkpoint pathway (Foley and Kapoor, 2013; Etemad and Kops, 2016).

The structure of kinetochore is conserved in eukaryotic species, despite the divergence of its subunits in the primary sequence (Venkei et al., 2012; Liu et al., 2016). The Spc105p protein is a crucial member of the kinetochore protein family in *Saccharomyces cerevisiae* and is responsible for correctly binding the kinetochore to spindle microtubules, as well as recruiting the checkpoint proteins (Pagliuca et al., 2009). In *Schizosaccharomyces pombe*, the Spc105p ortholog Spc7 has been implicated in spindle integrity and spindle checkpoint signaling (Kerres et al., 2007; Meadows et al., 2011). The segregation of chromosomes determines the development of *Aspergilli* to a large extent, and a loss of certain genes related to chromosome segregation greatly influences vegetable growth (Herrero et al., 2011; Kokkelink et al., 2011). Spc105, the unique ortholog of *S. cerevisiae* Spc105p in *A. flavus*, is a putative chromosome segregation protein and has not yet been investigated to our knowledge.

In this study, we demonstrate for the first time that the conserved kinetochore protein Spc105 in *A. flavus* regulates developmental differentiation, including conidia development and sclerotia production, and secondary metabolism. We further showed that Spc105 interacts directly with the global regulator LaeA, and that a functional interdependence exists between the two proteins. The relationship between Spc105 and LaeA highlights the coordinated interplay between distinct regulators, ensuring precise fungal development and secondary metabolism.

## Materials and Methods

### Strains and Culture Conditions

The *A. flavus* strains used in this study are listed in Supplementary Table S1 and were maintained as glycerol stocks. All strains were grown on potato dextrose agar (PDA, Difco) and was supplemented with 0.5 mg/ml uracil and 1.26 mg/ml uridine when necessary. For *A. flavus* transformation, strains were cultured on glucose minimal medium (GMM) with appropriate supplements for spore collection at 30°C for 7 days. For yeast transformation, *S. cerevisiae* Y2HGold strain was cultured on yeast extract peptone dextrose (YPD) plates at 30°C for 3 days.

### Strain Construction

*A. flavus* NRRL3357 (kindly provided by Gary Payne) was used as the wild-type strain, and *A. flavus* NRRL3357-5 (He et al., 2007), was used for *spc105* gene disruption or overexpression. All primers used for strain construction are listed in Supplementary Table S2. A fusion PCR based strategy was used for construction of *spc105* deletion and overexpression mutants (Szewczyk et al., 2006). Due to the lethality of entire *spc105* (GenBank accession number: AFLA_087770, AspGD accession number: AFL2T_01438) ORF deletion, we created the half of the *spc105* ORF (2534–4852 nt) deletion mutants (Supplementary Figure S1A). Briefly, the 1.2 kb 5′ and 1.2 kb 3′ flank regions of *spc105* ORF (2534–4852 nt) were amplified with primers *spc*/5F, *spc*/5R and *spc*/3F, *spc*/3R. The 1.9 kb selection marker *pyrG* gene were amplified from *Aspergillus fumigatus* genomic DNA with primers *pyrG*/F and *pyrG*/R. Primers *spc*/NF and *spc*/NR were used to amplify the 4.2 kb fusion construct by double joint PCR. To construct the *spc105* overexpression strain, the *A. nidulans* glyceraldehyde-3-phosphate dehydrogenase gene (*gpdA*) promoter was amplified from the *A. nidulans* genome using primers *gpdA*/F and *gpdA*/R and was fused upstream of the *spc105* coding region. Then the *spc105* 5′ flank, *A. fumigatus pyrG*, and *gpdA*-*spc105* fragments were joined together (Supplementary Figure S1A). Putative transformants were analyzed by Southern blot, diagnostic PCR, reverse transcription PCR (RT-PCR) and quantitative RT-PCR (qRT-PCR) (Supplementary Figure S1B). To determine the localization of Spc105 in *A. flavus*, an eGFP tag amplified from the pEGFP-C1 vector was inserted at the N-terminus of the Spc105 protein, and driven by the *gpdA* promoter with a five Gly-plus-Ala repeat (GA-5) linker (Yang et al., 2004). The above PCR constructs were transformed individually into *A. flavus* NRRL3357-5 protoplasts based on the polyethylene glycol method (Oakley et al., 2006). For complementation of *spc105* deletion mutant, a 1.5 kb of *spc105* upstream sequence and 2.3 kb of the *spc105* partial ORF were fused together. The fusion PCR fragments were digested with *Hin*dIII and *Kpn*I and then cloned into the pPTRI vector (Takara, Japan) harboring the pyrithiamine (PT) resistance gene. The confirmed recombined vector pPTR-*spc105* was transformed into Δ*spc105* protoplasts. All transformants were analyzed by PCR and qRT-PCR (Supplementary Figures S1C–E).

To induce *laeA* expression, the PCR fusion fragments of the *gpdA* promoter to the *laeA* ORF were digested with *Hin*dIII and *Kpn*I and then cloned into the pPTRI vector. The confirmed recombined vector was transformed into Δ*spc105* and *A. flavus* NRRL3357 protoplasts. For *laeA* gene deletion, the 5′ and 3′ flank regions of *laeA* were amplified and fused with the *ptrA* gene, which was amplified from the pPTRI vector. The deletion construct was introduced into the *OE:spc105* and *A. flavus* NRRL3357 strains. Transformants were selected on GMM containing 0.1 μg/ml pyrithiamine (PT, Sigma) and were confirmed by PCR and qRT-PCR analysis (Supplementary Figure S1).

To generate C-terminal HA-tagged LaeA and N-terminal 3xflag tagged Spc105 strains, *A. flavus* TXZ 21.3 (Δ*ku70*, Δ*argB*, *pyrG*-) was used (Zhao et al., 2017). Briefly, the HA tag sequence was inserted before *laeA* termination codon through fusion PCR and the *laeA*-HA-3′ flank region PCR fragment was generated. Then, the *laeA* 5′ flank, *argB* marker and *gpdA* promoter were amplified and fused with the above fragment. The PCR fragment was transformed into TXZ 21.3 protoplast, and transformants were selected and confirmed (data not shown). One of the resulting mutants, called LH-1, was used as the parental strain for the following transformation. Next, we fused the 3xflag sequence at the N-terminal of *spc105* ORF. The *pyrG* marker was used and the similar fusion PCR strategy was conducted to generate the final fusion fragments which were then transformed into LH-1 protoplast.

### Fungal Physiology Experiments

For morphological observation of colonies, 1 μl of a conidia suspension containing approximately 10^3^ conidia was point inoculated on PDA, GMM, and Czapek’ medium (CZ, Difco) solid plates and cultured in the dark for 7 days. For quantitative analysis of conidial production, PDA plates were overlaid with 5 ml of a suspension of conidia (10^6^ spores/ml) in 0.7% molten agar. Conidia were harvested from three 7 mm cores which were individually homogenized in 0.05% Triton X-100 solution and quantified with a hemocytometer. Sclerotia production was measured as previously described (Zhi et al., 2017) by counting sclerotia from CZ culture plates after incubation for 14 days at 30°C in the dark.

### Microscopic Analysis and Nuclear Staining

For the conidia germination assay, *A. flavus* conidia were inoculated in 5 ml potato dextrose broth (PDB, Difco) liquid media with coverslips at 37 and 25°C. The morphology of germinated conidia and hyphae were observed using a light microscope at different time intervals. For examination of nuclear division, samples were fixed with an appropriate fixing solution for 10 min (Ahn et al., 2014). They were washed twice in distilled water and stained in 100 ng/ml 4′,6-diamidino-2-phenylindole (DAPI, Sigma). Samples were then washed twice in phosphate-buffered saline (PBS) and viewed using a Leica fluorescence microscope.

### Examination of AF, Kojic Acid, and CPA Production

Aflatoxin B_1_ (AFB_1_) production were measured using modified thin layer chromatography (TLC) analysis as previously described (Lin et al., 2013). Each strain was inoculated in 30 ml PDB at 30 and 25°C respectively, and the mycelia and cultures parts were collected at different time points for AFB_1_ extraction. The extract residue was resuspended in acetone and developed on Si250 silica gel plates with chloroform-acetone (9:1, v/v). The plates were visualized under 254 nm UV light. Standard AFB_1_ was purchased from Sigma.

Kojic acid production was determined using the colorimetric method (Bentley, 1957). Briefly, *A. flavus* strains were cultured on PDA supplemented with 1 mM FeCl_3_ for 7 days at 30 and 25°C respectively. Kojic acid forms a chelated compound with ferric ions and subsequently generates a red color, allowing for a qualitative comparison between different strains.

CPA production was detected using TLC (Duran et al., 2007). *A. flavus* strains were inoculated into 50 ml PDB medium and incubated in the dark at 30 or 25°C for 7 days. Each culture broth was filtered through filter paper and subsequently extracted with chloroform for three times. The TLC plate was sprayed with a 2% solution of oxalic acid in methanol followed by heating in a dry oven at 85°C for 15 min. Toluene–ethyl acetate–acetic acid (8:1:1, v/v/v) was used as the developing solvent. All the above experiments were performed with four replicates.

HPLC analysis of the above three secondary metabolites was performed using a Shimadzu LC-20AT system (Shimadzu). Extracts of each sample were separated on a Luna C18 column (Phenomenex) which was equilibrated with a running solvent consisting of acetonitrile–water (36:65) for AF detection, methanol–water (10:9) for kojic acid detection, and acetonitrile–0.1% trifluoroacetic acid (50:50) for CPA production.

### Peanut Infection Assay

Peanut cotyledon colonization assay was performed as described previously (Kale et al., 2008). Surface sterilized peanut cotyledons were inoculated with a 10^5^ spores/ml of *A. flavus* and incubated for 3 days at 30°C in dark conditions. The infected seeds were collected in 50 ml Falcon tubes and mixed with 10 ml sterile 0.05% Triton X -100, followed by 1 min vortex to release the spores. The spores were diluted and counted using a hemocytometer. AFB_1_ production detection was performed by adding 10 m chloroform to the Falcon tubes, followed by shaking for 5 min three times.

### qRT-PCR Analysis

For detection of AF biosynthesis related gene expressions, spores were inoculated to 30 ml PDB to a final concentration of 2 × 10^5^/ml and incubated at 25°C and 30°C with shaking (200 rpm) for 48 h. Total RNA was extracted from the harvested mycelia using Trizol Reagent (Invitrogen, Carlsbad, United States) and cDNA was synthesized from 1 μg RNA using the HiScript α Q RT SuperMix cDNA Synthesis kit (Vazyme, Nanjing, China). qRT-PCR assay was performed using the Applied Biosystems Step One Plus system (Invitrogen) with SYBR Green detection as described previously (Zhi et al., 2017). Gene expression levels were normalized (2^–ΔΔCt^ analysis) to *A. flavus* β-actin gene expression levels. All analyses were performed in triplicate.

### RNA Sequencing and Data Analysis

RNA samples from three *A. flavus* independent biological repeats were prepared. Strains were grown in PDB at 25°C for 48 h and mycelia were harvested immediately for RNA extraction using Trizol Reagent (Invitrogen). The quality and quantity of isolated RNA were determined using an Agilent 2100 bioanalyzer system and RNA integrity numbers (RINs) were calculated. RNA samples with an RIN ≥ 8 were used for sequencing libraries preparation with an Illumina TruSeq RNA Sequencing Kit. The libraries were sequenced on an Illumina Hiseq2500 system (Oebiotech, Shanghai, China).

Raw sequencing reads obtained for each sample were quality controlled using FastQC (Andrews, 2010) and then filtered to remove the low-quality reads using version 2.3.3 of the NGS QC TOOLKIT (Patel and Jain, 2012). The remaining reads were mapped to the *A. flavus* NRRL3357 genome (GCF_000006275.2) using version 2.0.13 of tophat2 (Kim et al., 2013) and version 2.2.4 of bowtie2 (Langmead and Salzberg, 2012). Gene expression level was normalized by calculating the number of Fragments Per Kilobase per Million reads mapped (FPKM) (Trapnell et al., 2010), and the base mean of each gene from the six sequenced samples was analyzed using DESeq software (Anders and Huber, 2012) to identify DEGs. Genes with |log2 (fold change)| ≥ 1.5 and adjusted *P*-value (padj) ≤ 0.01 were defined as significantly differentially expressed. The gene-function annotation was conducted based on the GO and KEGG databases (Kanehisa et al., 2008).

### Yeast Two-Hybrid Assay

Y2H assay was performed using the Matchmaker Gold Y2H System (Clontech), according to the manufacturer’s instructions. The *spc105* and *laeA* inserts were amplified from *A. flavus* cDNA templates and cloned into pGADT7 and pGBKT7, respectively. The sequences of all inserts were verified by DNA sequencing analysis. Recombined vectors were transformed into the *S. cerevisiae* Y2HGold strain and plated onto selective media (SD/-Leu/-Trp) at 30°C for 3 days. Then, the transformants were plated on the selective synthetic dextrose medium (SD/-Leu/-Trp/-His/-Ade/ + X-α-gal) and incubated at 30°C for 3–5 days.

### Recombinant Protein Purification and GST Pull-Down Assay

Heterologous expression and subsequent purification of Spc105 and LaeA proteins were conducted in *E. coli* BL21 (λDE3) using a combination of GST fusion vector pGEX4T-1 (GE Healthcare) and N-terminal 6X histidine-tag fusion vector pET28a. Briefly, the *spc105* gene was inserted into pGEX4T-1 to express GST-Spc105, which was subsequently purified on glutathione-agarose 4B (GE Healthcare) following manufacturer’s recommendations. The *laeA* gene was cloned into pET28a to yield a His_6_-LaeA fusion protein, which was purified using Ni-NTA agarose (GE Healthcare).

GST pull-down experiments were performed according to the manufacturer’s recommendations. Briefly, 20 μg purified GST-Spc105 protein, 25 μl glutathione magnetic beads (Pierce), and 20 μg purified His_6_-LaeA protein were co-incubated for 3 h at 4°C in PBS buffer. The magnetic beads were subsequently washed 6 times with PBS buffer and boiled for 10 min in SDS-PAGE loading sample buffer. SDS-PAGE and Western blot analysis were subsequently conducted. Immunodetection of His_6_-LaeA was performed using a Mouse His-tag monoclonal antibody at a dilution of 1:5000 (Proteintech) followed by Goat anti-Mouse HRP 1:10,000 (Proteintech).

### Co-immunoprecipitation Assay

To perform co-immunoprecipitation (Co-IP) assay, C-terminal HA-tagged LaeA and N-terminal 3xflag tagged Spc105 strains were generated. The strains were grown in PDB for 24 h at 30°C, and mycelia were collected and ground to a fine powder with liquid nitrogen. Each sample was resuspended in 1 ml of IP lysis buffer (Pierce), and centrifuged at 16,000 × *g* at 4°C for 15 min. The supernatant was collected, and the protein content was measured using a BCA protein assay kit (Pierce). The same amount of protein for each sample was added to 40 μl of Dynabeads Protein G (Thermo Fisher Scientific) previously incubated with monoclonal anti-Flag antibody (Sigma). The beads was washed three times with PBST (0.02% Tween) prior to incubation. Cell extracts and beads were then incubated with shaking at 4°C for 12 h. After incubation, the beads were washed three times in PBST (0.02% Tween) by placing the tube in a DynaMag2 magnet. Samples were incubated with Sample Buffer and boiled at 100°C for 5 min. Proteins were transferred from a 10% SDS-PAGE gel onto a PVDF membrane for Western blotting. HA-tagged LaeA was detected using a mouse anti-HA antibody (MBL) at 1:5,000 dilution and a goat anti-mouse IgG horseradish peroxidase (HRP) antibody (Proteintech) at 1:5,000 dilution. Signal was detected by ECL with GBOX-CHENI (Syngene).

### Statistical Analysis

All statistical analyses were performed using GraphPad Prism (version 5.0; GraphPad Software) and *P* < 0.05 was considered a significant difference.

## Results

### Preliminary Characterization of *A. flavus* Spc105

**FIGURE 1:**
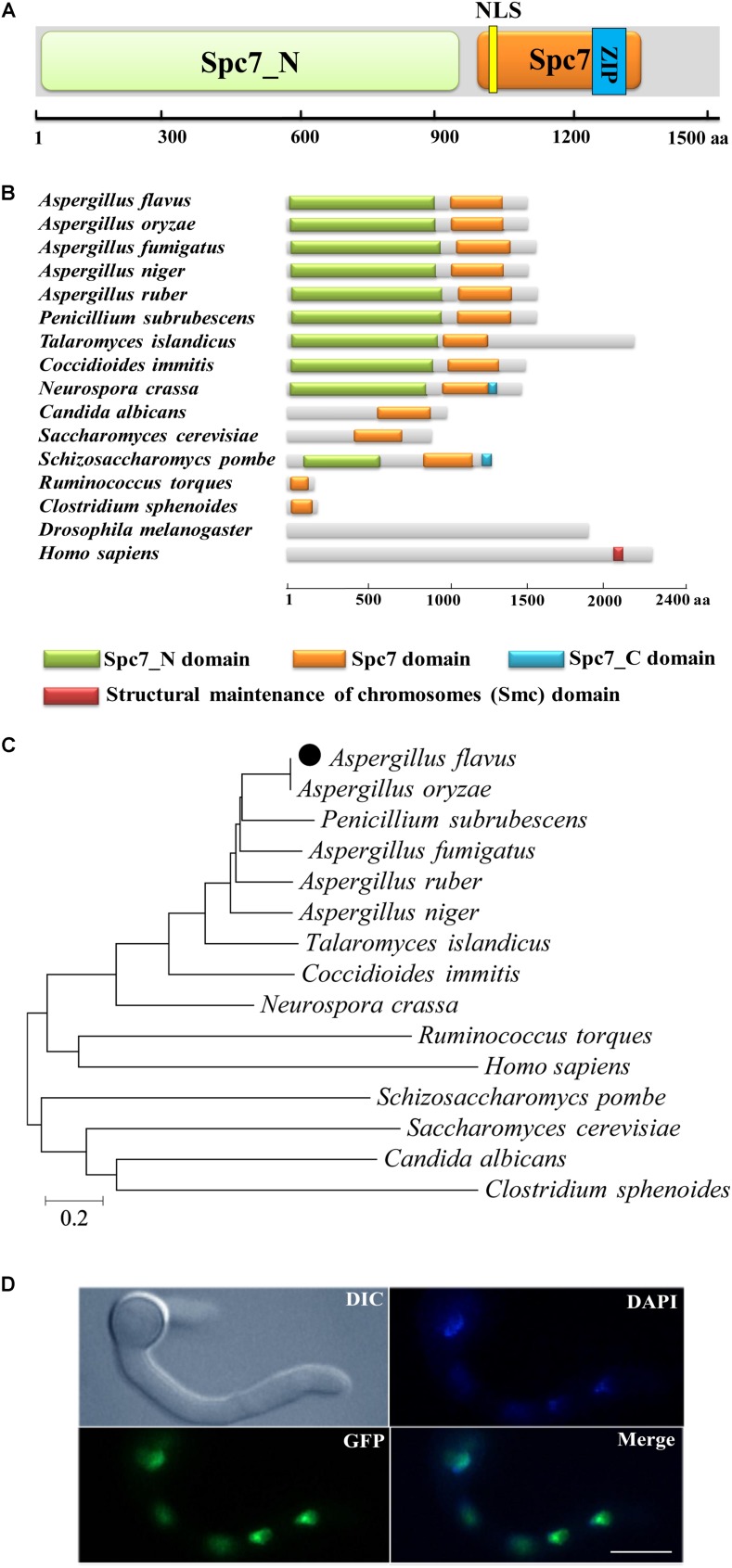
Preliminary characterization of *A. flavus* Spc105. **(A)** Graphical representation of *A. flavus* Spc105 protein. NLS, bipartite nuclear localization signal; ZIP, leucine zipper motif. **(B)** Domain analysis of Spc105 protein between different species. **(C)** Phylogenetic analysis of Spc105 proteins. A neighbor-joining phylogenetic tree was constructed based on sequence alignments of Spc105 proteins using ClustalX2. **(D)** Localization of Spc105 in *A. flavus*. GFP was fused to the N-terminal of Spc105. Nuclei were stained with 100 ng/ml of 4′,6-diamidino-2-phenylindole (DAPI) and examined by fluorescence microscope.

The *A. flavus spc105* (AFL2T_01438) ORF was predicted to consist of 4,852 nucleotides, with four introns, and encodes a putative chromosome segregation protein (Spc105) containing 1541 amino acids (aa). The predicted *A. flavus* Spc105 that harbors a Spc7 (the Spc105p ortholog in *S. pombe*) kinetochore protein domain (residues 1005–1328 aa), and a Spc7_N domain (residues 32–940 aa) was 26% and 31% identical to *S. cerevisiae* Spc105p and *S. pombe* Spc7, respectively (Figure 1A). A bipartite nuclear localization signal (residues 1031–1046 aa) and a leucine zipper motif (residues 1169–1252 aa), which might mediate protein-protein interactions, were predicted by the program PROSITE (Sigrist et al., 2010). The structural analysis of Spc105 proteins from several species showed that all analyzed fungi and bacteria share a conserved Spc7 domain (Figure 1B), and Spc105 proteins from *Aspergillus* most closely resemble their orthologs in *Penicillium subrubescens* (Figure 1C).

Through green fluorescent protein (GFP) labeling we found that the Spc105 protein is localized in the nucleus of *A. flavus* (Figure 1D). qRT-PCR analysis of *spc105* gene expression in a wild-type (WT) strain showed that it is a low-expression-level gene, and its expression was almost constant during the vegetative growth phase (data not shown), implying that *spc105* is a constitutively expressed gene in *A*. *flavus*.

### Spc105 Influences Colony Growth and Conidia Germination

**FIGURE 2:**
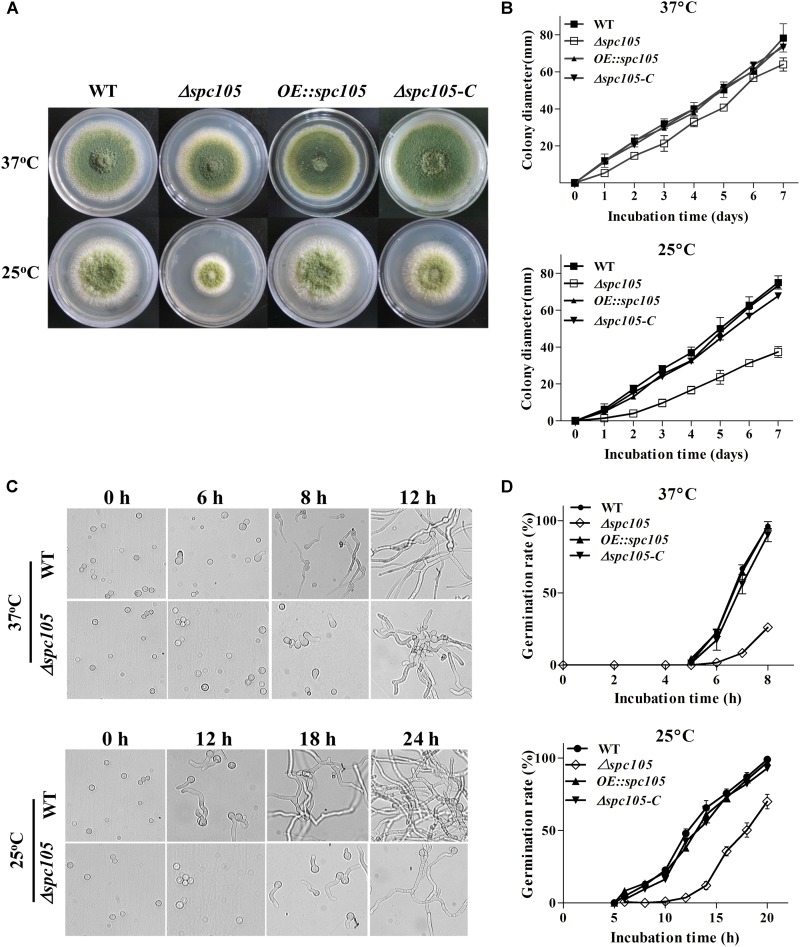
Spc105 affects colony growth and spore germination of *A. flavus*. **(A)** Colony growth of *spc105* mutants on PDA plates after 5 days incubation. **(B)** Colony diameter measurement of each strain. **(C)** Examination of spore germination in PDB culture. **(D)** Comparison of germination rate of each strain.

To investigate the role of Spc105 in *A. flavus*, we first attempted to completely delete *spc105* gene, however, it was unsuccessful. Considering that the mutation of RVSF motif in Spc105 N-terminal is lethal in *S. cerevisiae* (Rosenberg et al., 2011), and *A. flavus* Spc105 also contains an RVSF motif (202–205 aa), we speculated that *spc105* is an essential gene for the viability of *A. flavus*. Thus, we created *spc105* gene partial ORF (2534–4852 nt) deletion (*Δspc105*), *spc105* gene overexpression (*OE:spc105*) and complementation (*Δspc105*-*C*) strains (Supplementary Figure S1). The *Δspc105* strain displayed inhibited colony growth on all tested culture plates including potato dextrose agar (PDA), glucose minimal media (GMM), and Czapek’ media (CZ) (Figure 2 and data not shown). This inhibitory effect was stronger at 25°C than at 37°C compared to the WT, *OE:spc105*, and *Δspc105*-*C* strains. Moreover, conidia germination of the *Δspc105* strain was obviously delayed, especially at 25°C (Figure 2). There was no conidial swelling for up to 10 h in the *Δspc105* strain at 25°C. After 18 h incubation, conidia of the WT, *OE:spc105*, and *Δspc105*-*C* strains had germinated almost 100% while only about 60% of the *Δspc105* conidia had germinated at 25°C. Surprisingly, although the colony growth and germination rates of the *Δspc105* strain were both delayed, the total mycelia mass in liquid culture was not significantly different from the WT after 24 h incubation (Supplementary Figure S2). These results suggest that the *Δspc105* strain is cold-sensitive and Spc105 affects normal colony growth and conidia germination in *A. flavus*.

### Spc105 Regulates Conidiophore Development and Sclerotia Production

**FIGURE 3:**
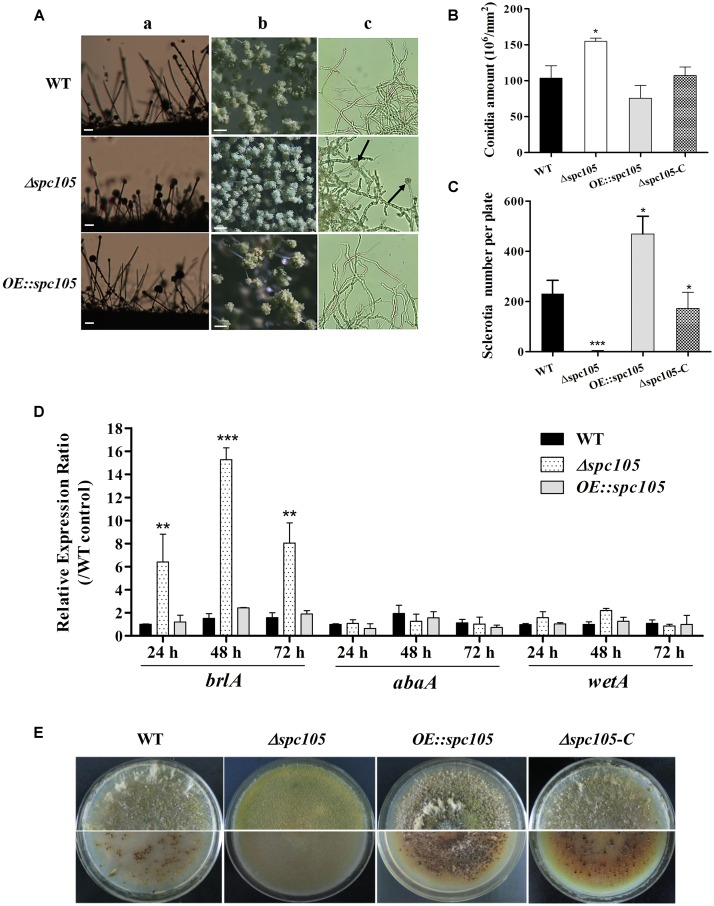
Spc105 regulates conidia development and sclerotia formation in *A. flavus*. **(A)** Conidia development of each strain cultured at 30°C. a, microscopic observation of conidiophore formation after 24 h incubation on PDA plates. b, examination of conidial head structure under a stereomicroscope after 48 h incubation on PDA plates. Bars represent 200 μm. c, microscopic examination of conidiophore formation after 48 h incubation in submerged PDB culture. Arrows indicate the degenerate conidiophores. **(B)** Quantitative analysis of conidial production on PDA plates. **(C)** Statistic analysis of sclerotia production. **(D)** Relative gene expressions of *brlA*, *abaA*, and *wetA*. Strains were cultured in PDB and gene expression levels were normalized (2^–ΔΔCT^ analysis) to *A. flavus* β-actin gene expression levels. **(E)** Observation of sclerotia formation. Each strain was grown on CZ agar plates for 2 weeks in the dark at 30°C. ^*^P < 0.05; ^∗∗^*P* < 0.01; ^∗∗∗^*P* < 0.001.

Microscopic observations revealed that *spc105* deletion resulted in preferential development of conidiophore in *A. flavus*. The *Δspc105* strain produced denser, smaller conidial heads compared with those of WT, *OE:spc105*, and *Δspc105*-*C* strains (Figure 3A). Conidiophore stipes were slightly shorter in the *Δspc105* strain, resulting in a somewhat flat colony phenotype in contrast to the typical floccose appearance of WT strains (Figure 3A). Moreover, the *Δspc105* strain produced degenerate conidiophores in submerged culture, a condition which normally completely blocks asexual development (Han et al., 2001), while no conidiophores were observed in the WT, *OE:spc105*, and *Δspc105*-*C* strains (Figure 3A and data not shown). Further quantitative analysis of conidial production showed a twofold increase in the amount of conidia in the *Δspc105* strain and a slight reduction in the *OE:spc105* strain compared to WT (Figure 3B). qRT-PCR analysis of the conidial development regulatory genes revealed that *brlA* transcription level was remarkably increased in *Δspc105* strain during vegetative growth in liquid culture (Figure 3D), whereas *abaA* and *wetA* gene expressions were not significantly different from that in WT and *OE:spc105* strains.

Additionally, *spc105* deletion abolished the production of the resistant structure sclerotia while *spc105* overexpression enhanced sclerotia production (Figure 3E). No sclerotia were formed in the *Δspc105* strain compared with the WT (230 ± 20), *OE:spc105* strains (469 ± 21), and *Δspc105*-*C* (188 ± 15) after 14 days of incubation in the dark on sclerotia conducive CZ plates (Figure 3C). The above results suggest that Spc105 regulates conidia development and sclerotia formation in *A. flavus*.

### Deletion of *spc105* Delays the Nuclear Division Cycle

**FIGURE 4:**
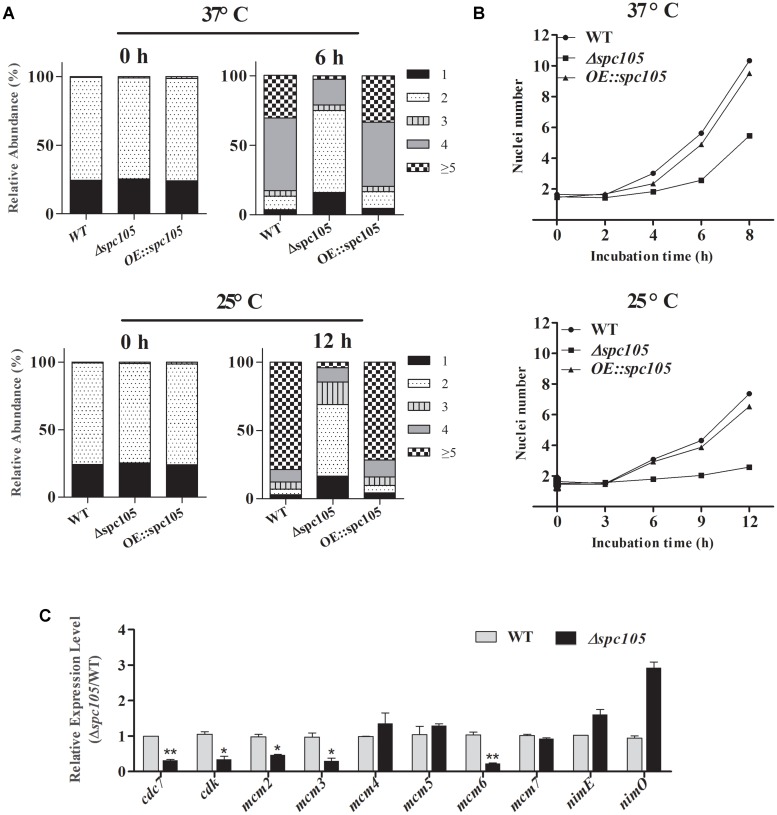
Involvement of Spc105 in the nuclear division of *A. flavus*. **(A)** Graphic image of the counted nuclei, the numbers 1, 2, 3, 4, and ≥5 represent the nuclei number. **(B)** The mean number of nuclei per germling of each strain. **(C)** qRT-PCR analysis of meiosis-related gene expression. Strains were incubated for 12 h in 100 ml PDB at 25°C and mycelia samples were collected for RNA extraction. Relative expression ratios were calculated relative to the WT control. Error bars represent the standard deviation based on three replicates. ^*^P < 0.05; ^∗∗^*P* < 0.01.

DAPI staining of nuclei during conidia germination revealed that *spc105* deletion delayed the nuclear division cycle in *A. flavus* (Figure 4). When cultured in PDB at 37°C, the majority (approximately 70%) of WT and *OE:spc105* conidia had undergone the first round of nuclear division within 4 h after inoculation. When incubated for 6 h, approximately half of the WT and *OE:spc105* germlings had 4 nuclei, while 70% of the *Δspc105* strain conidia had still one or two nuclei (Figure 4A). At 25°C, the number of nuclei increased slowly as a function of time. Up to 12 h, more than 5 nuclei were observed in most of the WT and *OE:spc105* germlings which suggest that 3 or 4 round of nuclear division had completed. In contrast, most of the *Δspc105* conidia were still uninucleate or binucleate (Figure 4A). Figure 4B shows the mean number of nuclei per germling at each time point. The results indicated that *spc105* deletion in *A. flavus* caused nuclei to transiently arrest in interphase, although nuclear division was eventually completed in the *Δspc105* strain. Further examination of the expression of several mitosis-related genes (Yeeles et al., 2015) revealed that the expression levels of *cdc7* (cell division cycle 7), *cdk* (cyclin-dependent kinase A), and three (*mcm2*, *mcm3*, and *mcm6*) out of the six *mcm* (minichromosome maintenance proteins 2–7) genes were downregulated in *Δspc105* strain. Alternatively, the expression of *nimE* (cyclin B) and *nimO* (dbf4 kinase) did not show significant differences (Figure 4C). These results confirmed that Spc105 is involved in the nuclear division of *A. flavus*.

### Spc105 Affects Secondary Metabolite Production and Colonization of *A. flavus*

**FIGURE 5:**
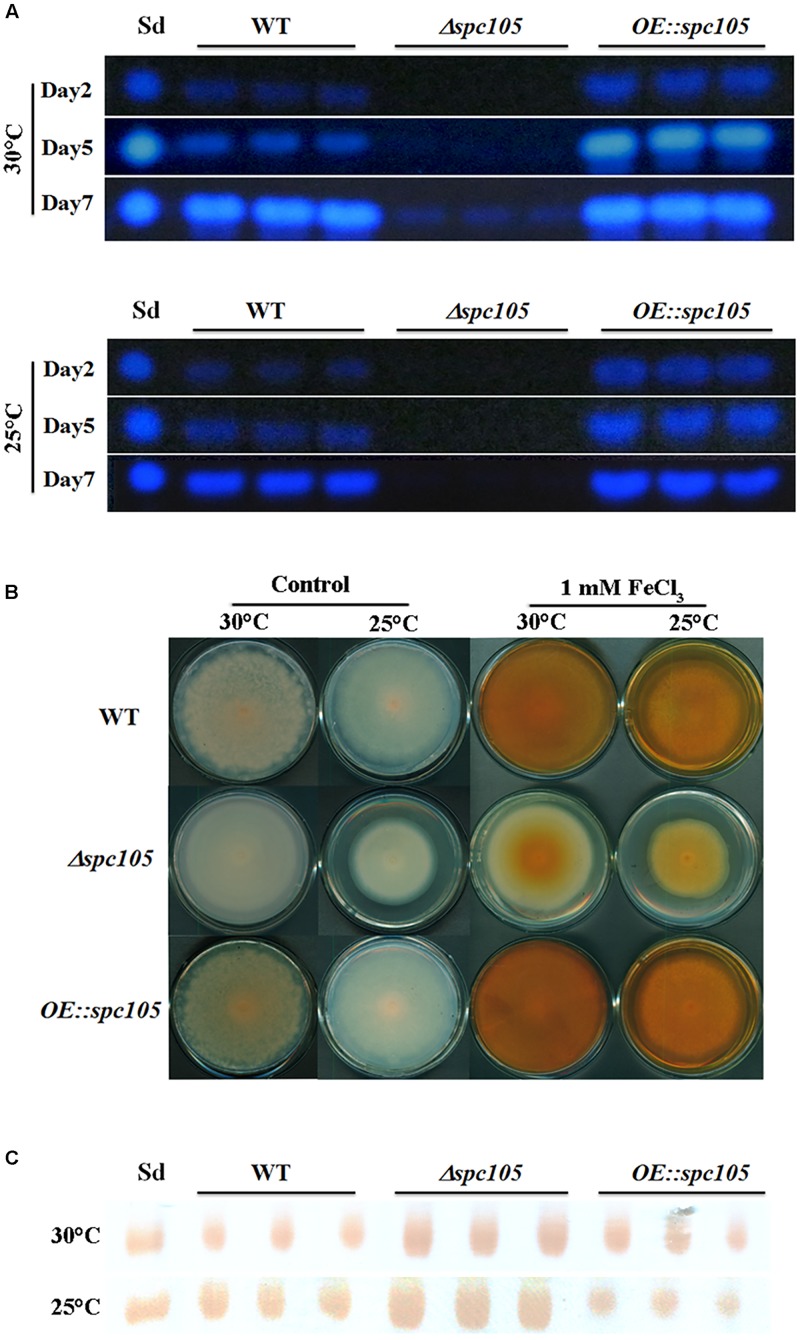
Spc105 regulates the production of aflatoxin, kojic acid, and cyclopiazonic acid. **(A)** Time course semi-quantitative thin-layer chromatography (TLC) analyses of aflatoxin B_1_ (AFB_1_) production in the PDB culture. Sd represents the AFB_1_ standard. **(B)** Determination of kojic acid production in solid medium through the colorimetric method. Strains were incubated on PDA plates for 7 days. Images are representative of four experimental replicates. **(C)** TLC detection of CPA production in PDB liquid culture of each strain. Sd represents the CPA standard.

*A*. *flavus* contains 56 secondary metabolite clusters in its genome and produces a wide variety of secondary metabolites, the biosynthesis of which is often associated with morphological differentiation (Bayram and Braus, 2012). Aflatoxin B_1_ (AFB_1_) is the most crucial and abundant metabolite in *A. flavus*. Detection of both the culture and mycelia extracts showed that *spc105* deletion almost completely abolished AFB_1_ production while *spc105* overexpression resulted in increased AFB_1_ levels (Figure 5A), suggesting a significant positive effect of Spc105 on AF production. Furthermore, the AF production of *Δspc105*-*C* strain was comparable to WT (Supplementary Figure S3A). The production of kojic acid, an important chemical material, was also positively affected by Spc105 (Figure 5B). In addition, the production of the mycotoxin CPA was increased in the *Δspc105* strain and slightly reduced in the *OE:spc105* strain, compared with WT (Figure 5C). The above results were confirmed by high-performance liquid chromatography (HPLC) analysis (Supplementary Figure S4). All analyses indicated that Spc105 exerts a role in *A*. *flavus* secondary metabolite biosynthesis.

**FIGURE 6:**
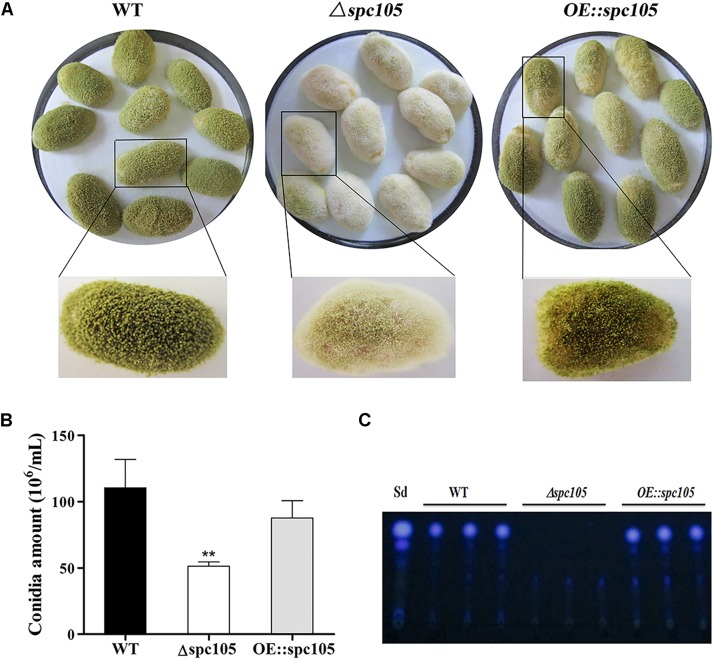
Growth and aflatoxin production of WT, *Δspc105*, and *OE:spc105* strains on peanut seeds. **(A)** Photographs of peanut seeds infected with *A. flavus* strains after 3 days of incubation at 30°C. **(B)** Quantification of conidia from the infected peanut seeds by *A. flavus* strain. ^∗∗^*P* < 0.01. **(C)** TLC analysis of AFB_1_ levels in infected peanuts.

In addition, surface-sterilized peanut seeds were inoculated with the above three *A. flavus* strains to assess the host colonization capability of *spc105* mutants. Results showed that the *Δspc105* strain grew less vigorously on peanuts than WT, *OE:spc105*, and *Δspc105*-*C* strains (Figure 6 and Supplementary Figure S3), corresponding to a significant decrease in the conidial production on seeds (Figure 6). Moreover, AFB_1_ was not detected in the *Δspc105* infected seeds (Figure 6).

### Transcriptome Comparison Between *Δspc105* and WT

Differential gene expression analysis, carried out by assessment of the genome wide transcriptional profile between *Δspc105* and WT strains cultured at 25°C for 48 h, identified 1,846 differentially expressed genes (DEGs), including 670 upregulated and 1,176 downregulated genes (Supplementary Figure S5).

Functional analysis based on Gene Ontology (GO) and Kyoto Encyclopedia of Genes and Genomes (KEGG) pathway annotation revealed that the downregulated genes were involved in various cellular processes mainly associated with AF biosynthesis, fatty acid metabolism, rRNA processing, and the ribosome biogenesis-related process (Supplementary Figure S6). The significant downregulation of AF biosynthetic genes in *Δspc105* was consistent with the above AF detection results. Further, the upregulated gene set was mainly implicated in the process of carbohydrate metabolism, cell proliferation, protein phosphorylation, and ubiquitination. These results indicated that the absence of *spc105* had an extensive effect on the induction and repression of *A. flavus* genes.

**FIGURE 7:**
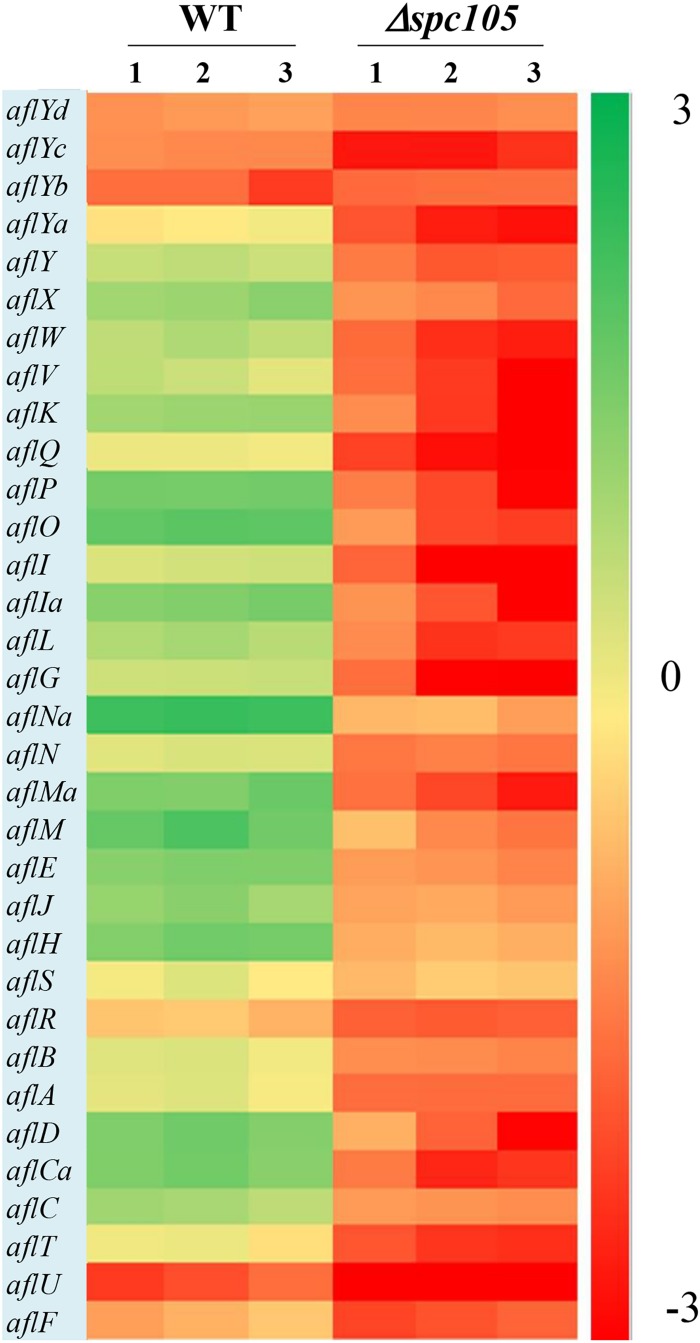
Gene expression analysis of AF cluster genes. The heatmap showed gene expression levels in Δ*spc105* and WT samples. Gene expression is plotted with colors corresponding to the log10 value of the FPKM values of each gene.

By focusing on the transcriptional changes in secondary metabolism genes induced by *spc105* deletion, we found that 23 out of the 74 backbone genes were differentially expressed, corresponding to 19 out of the predicted 56 secondary metabolite gene clusters, including the AF cluster (# 54), CPA cluster (# 55), and kojic acid cluster (# 56) (Supplementary Table S3). Among the 34 AF cluster genes, 31 genes were significantly downregulated in the *Δspc105* strain, including the AF cluster-specific regulatory genes *aflR* and *aflS* (Figure 7). These expression profiles were verified by qRT-PCR analysis (Supplementary Table S4).

In addition to the annotated AF cluster genes, several upstream regulatory genes were differentially expressed in our data, including the velvet complex encoding genes *laeA*, *velB*, and *velC* (another velvet family member) and the signal transduction related genes *pkaC* and *rasA* (Som and Kolaparthi, 1994; Table 1). These results may explain the observed phenotype and secondary metabolite production changes in the Δ*spc105* background.

**TABLE 1:** Effects of *spc105* deletion on regulatory gene expression in *A. flavus.*

	**FPKM value**					
					
**Gene name**	**Δ*spc105***	**WT**	**log2 fold change^a^**	***P*-value**	**Up/Down**	**Significant^b^**
*laeA* (AFLA_033290)	6.7729	24.5216	–1.9199	7.39E-06	Down	Yes
*veA* (AFLA_066460)	18.435	28.0173	–0.6892	0.14604	Down	No
*velB* (AFLA_081490)	83.8568	29.0156	1.7677	0.00012	Up	Yes
*velC* (AFLA_025780)	49.2205	12.0131	1.9301	1.66E-06	Up	Yes
*vosA* (AFLA_026900)	7.0229	8.6959	0.0284	0.82509	Up	No
*nsdC* (AFLA_131330)	59.9864	36.7608	0.7241	0.3363	Up	No
*nsdD* (AFLA_020210)	82.5784	156.0551	–0.7165	0.0529	Down	No
*pkaC* (AFLA_135040)	22.9050	8.2890	1.7552	4.21E-05	Up	Yes
*rasA* (AFLA_132380)	5.4108	26.2088	–2.0075	5.03E-05	Down	Yes

*^*a*^The mean of normalized counts in Δspc105/WT.^*b*^The genes with |Log2 fold change| ≥ 1.5 and P ≤ 0.01 were considered as significantly differentially expressed.*

### Interdependent Relationship Between Spc105 and LaeA

**FIGURE 8:**
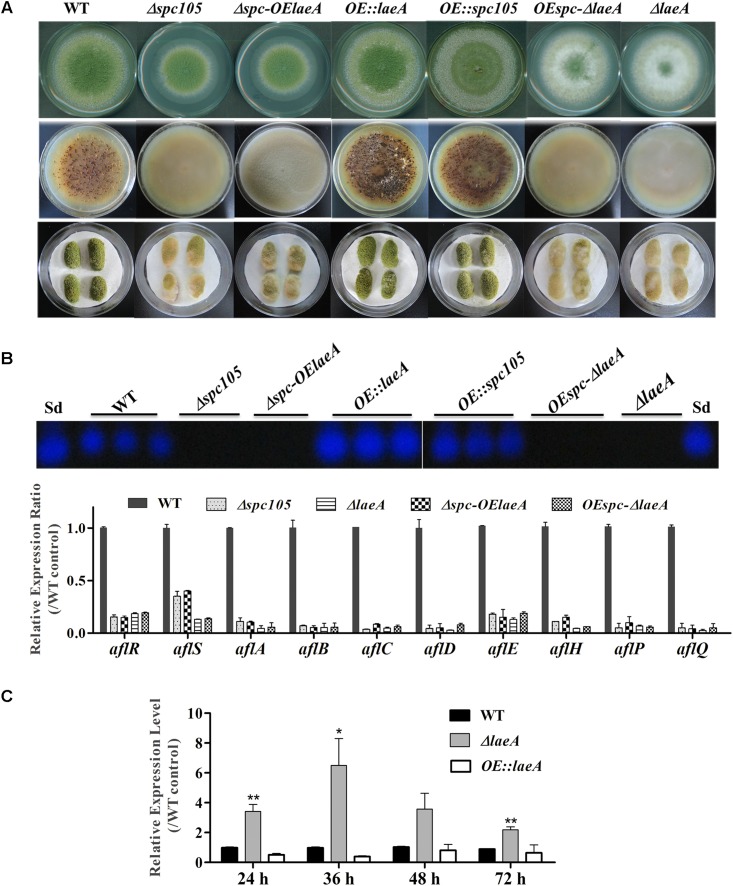
Relationship between Spc105 and LaeA. **(A)** Phenotypes of *spc105/laeA* double mutant strains. Upper: Phenotypes of mutant strains after 5 days incubation at 30°C on PDA plates. Middle: Sclerotia production of each strain on CZ plates after 2 weeks incubation in the dark. Bottom: Peanut infection of each strain after 3 days incubation at 30°C. **(B)** TLC analyses of AFB_1_ production of each strain and gene expression analysis of AF cluster genes in the PDB culture after 48 h incubation at 30°C. **(C)** Effect of *laeA* deletion and overexpression on *spc105* gene expression. Mycelia were harvested from PDB culture at the indicated time point. Error bars represent the standard deviations based on three replicates. ^*^P < 0.05; ^∗∗^*P* < 0.01.

Since *spc105* deletion reduced the expression of the global regulatory gene *laeA*, we hypothesized that Spc105 may correlate with LaeA to regulate secondary metabolism. To ascertain the potential functional relationship between Spc105 and LaeA, we generated Δ*spc*-*OElaeA* and *OEspc*-Δ*laeA* double mutant strains (Supplementary Figure S1). Results showed that fungal development, AF production, and AF cluster gene expression were similar between the Δ*spc*-*OElaeA* and Δ*spc105* strains (Figure 8 and Table 2), indicating that overexpression of *laeA* cannot restore the development and the AF production defects of the Δ*spc105* strain. Likewise, the *OEspc*-Δ*laeA* mutant exhibited properties like those of Δ*laeA*, suggesting that *spc105* overexpression failed to rescue the effects caused by the lack of *laeA* in *A. flavus*. We also observed that all above mutants displayed reduced expression levels of AF cluster genes, including *aflR* and *aflS*. These results suggested that Spc105 and LaeA hold interdependent functions. In addition, the transcription level of *spc105* was elevated in the Δ*laeA* background, where it was slightly decreased in the *OE:laeA* strain (Figure 8C), suggesting a negative regulatory effect of *laeA* on *spc105* gene transcription.

**TABLE 2:** Summary of *A. flavus spc105*/*laeA* double mutant phenotypes^a^.

**Parameters**	**WT**	**Δ*spc105***	**Δ*spc105-OElaeA***	***OE:laeA***	***OE:spc105***	***OEspc-*Δ*laeA***	**Δ*laeA***
Radial growth^b^	51 ± 1.5	35 ± 1.5	36 ± 1.2	51 ± 0.4	52 ± 1.0	51 ± 0.8	50 ± 1.2
Germination rate^c^	100%	50%	51%	100%	100%	100%	100%
Conidia production^d^	86 × 10^6^	155 × 10^6^	148 × 10^6^	75 × 10^6^	60 × 10^6^	45 × 10^6^	42 × 10^6^
Sclerotia production	230 ± 20	ND^e^	ND	505 ± 10	422 ± 21	ND	ND
Degenerate conidiophores formation in	ND	Yes	Yes	ND	ND	ND	ND
submerged culture							
Peanuts infection	Control	Reduced	Reduced	Enhanced	Enhanced	Reduced	Reduced

*^*a*^All strains were incubated at 30°C for phenotype observation. All analyses were performed in triplicate.^*b*^Radial growth on PDA plates after 5 days incubation, expressed in mm.^*c*^Germination rate was recorded after 10 h incubation in PDB culture when WT spores were 100% germinated.^*d*^Conidia amount per mm^2^ on PDA plates was recorded.^*e*^ND, not detected.*

### Spc105 Interacts Directly With LaeA

**FIGURE 9:**
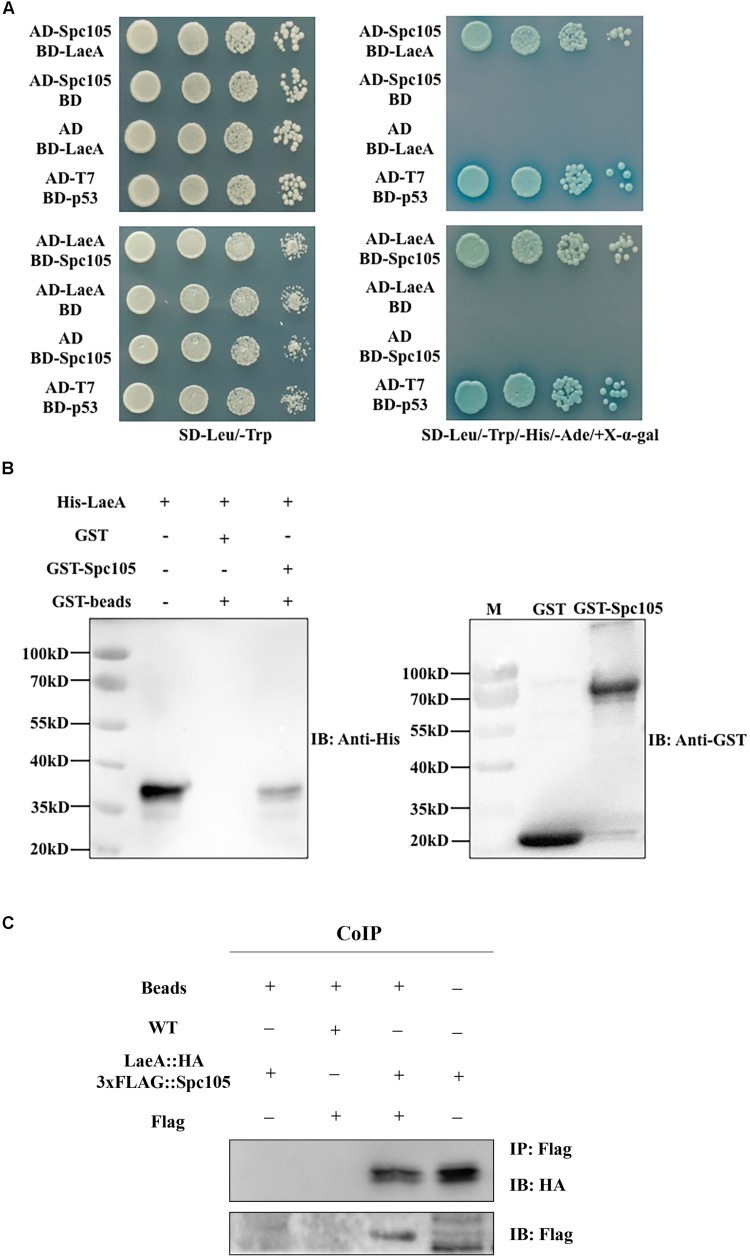
Spc105 interacts with LaeA in the Y2H and GST pull-down assays. **(A)** Y2H assays to determine protein–protein interactions. Yeast cells were grown in liquid selective medium overnight and diluted serially. Four microliters of serially diluted yeast cells were spotted on selective synthetic dropout media SD/-Leu/-Trp/-His/-Ade/ + X-α-gal and incubated at 30°C for 3–5 days. The SD/-Leu/-Trp plate is non-selective and served as the loading control. **(B)** GST pull-down assay of the interaction between Spc105 and LaeA *in vitro*. Recombinant GST and GST-Spc105 were incubated with recombinant His_6_-LaeA and subsequently purified by glutathione magnetic beads. Note that we tried to but failed to express the full length of recombinant GST-Spc105, and here is the truncated version (800 aa-1541 aa) of Spc105. Immunoblot analysis was performed to detect the presence of His_6_-LaeA using an anti-His-tag antibody. **(C)** Co-IP of LaeA:HA and 3xFLAG:Spc105. Affinity purification assays from Flag-tagged Spc 105 strain in the background of HA-tagged LaeA were performed with Flag-Trap magnetic beads. The coimmunoprecipitated proteins were analyzed by the anti-HA antibody.

The finding of functional interdependence between Spc105 and LaeA led us to hypothesize that Spc105 could directly interact with LaeA. To test this hypothesis, a yeast two-hybrid (Y2H) assay was performed and the results showed that the yeast cells co-transformed with AD-Spc105 and BD-LaeA plasmids exhibited positive galactosidase activity (Figure 9A). This result indicates that Spc105 directly interacts with LaeA. Similar results were obtained in yeast cells co-transformed with BD-Spc105 and AD-LaeA plasmids. The Spc105-LaeA interaction was also confirmed by the *in vitro* GST pull-down assay with recombinantly expressed GST-Spc105 and His_6_-LaeA (Figure 9B). Finally, we performed an *in vivo* co-immunoprecipitation (Co-IP) experiments using protein extracts from *A. flavus* strain expressing LaeA:HA and 3xFLAG:Spc105. Immunoprecipitates were analyzed by Western blotting with anti-HA antibody. As shown in Figure 9C, HA-LaeA was co-immunoprecipitated with Spc105 from lysates. Additionally, we observed negative results in examining whether Spc105 interacts with VelB or VeA, as determined by Y2H assay (data not shown).

**FIGURE 10:**
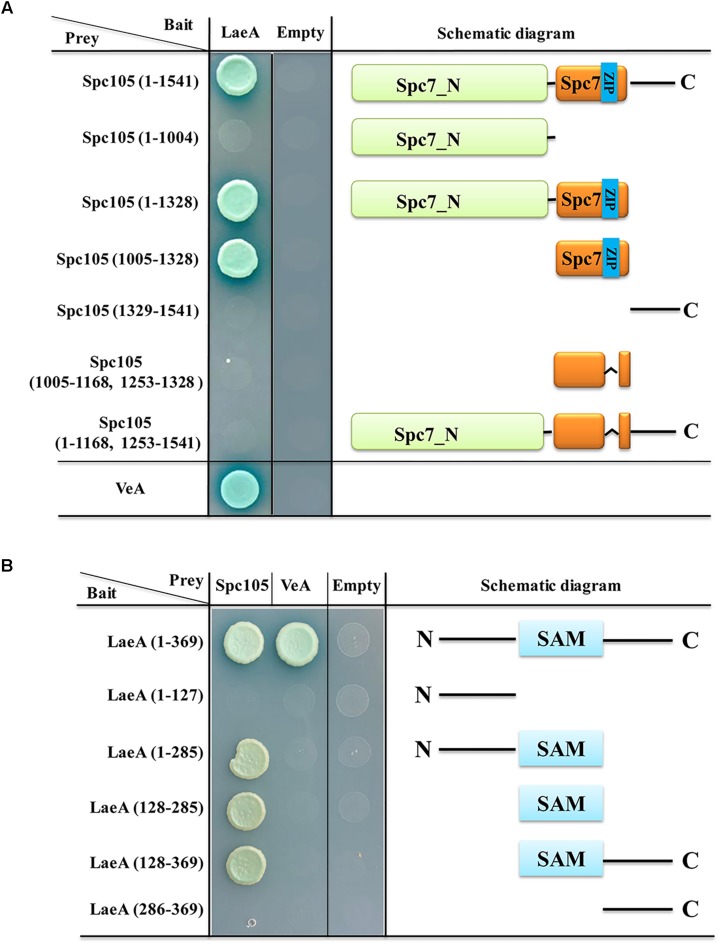
Interaction domain mapping of Spc105 and LaeA. **(A)** Six truncation mutants of Spc105 were constructed and tested against full length LaeA in the yeast-two-hybrid assay. **(B)** Five different truncations of LaeA were tested as interaction partners with full-length Spc105 and VeA.

LaeA is a constitutively nuclear protein and its interaction with VeA-VelB in the nucleus is required for the control of secondary metabolism and development (Bayram et al., 2008, 2010). Since both VeA and Spc105 can interact with LaeA, we investigated the interaction domains of LaeA-Spc105 and LaeA-VeA. Y2H assay of the interactions between full length LaeA with 6 different truncations of Spc105 revealed that Spc105-LaeA interaction depends on the Spc7 domain (1005–1328 aa), and that the leucine zipper (1169–1252 aa) in the Spc7 domain is required for this interaction (Figure 10A). Further, five different truncated forms of LaeA were tested as interaction partners with full length Spc105 and VeA. Our results showed that Spc105 can interact with the SAM binding domain (128–285 aa) of LaeA, whereas VeA can only interact with full length LaeA (Figure 10B). These results suggest that there may be a competition between the nuclear Spc105 and the light-sensitive protein VeA, which shuttles between the cytoplasm and nucleus.

## Discussion

Accurate chromosome segregation is crucial for cellular and organismal viability and requires coordinated interaction between the spindle and kinetochore (Etemad and Kops, 2016). As a key member of the kinetochore, the participation of Spc105 in the kinetochore-spindle interaction and the spindle assembly checkpoint (SAC) during chromosome segregation has been widely studied in model organisms (Aravamudhan et al., 2016; Liu et al., 2016). However, the role of the Spc105 orthologs in filamentous fungi has not been explored. In this study, we demonstrated that Spc105 in *A. flavus* interacts directly with LaeA, a global regulator of secondary metabolism, and plays multiple roles in developmental differentiation and secondary metabolism. To the best of our knowledge, this is the first report describing the function of the Spc105 ortholog in filamentous fungi, particularly in secondary metabolism.

Spc105 is functionally conserved in eukaryotes from yeast to humans. In human cells, a lack of hSPC105 activity results in lagging sister chromatids during metaphase and widespread chromosome mis-segregation at anaphase (Cheeseman et al., 2008). In *Drosophila melanogaster*, Spc105 inactivation completely abolishes the kinetochore-microtubule attachment (Feijão et al., 2013). In *Caenorhabditis elegans*, the RNAi-mediated depletion of the Spc105p ortholog KNL-1 prevents kinetochore assembly and leads to a kinetochore-null phenotype (Desai et al., 2003). In *S. pombe*, the Spc105p ortholog Spc7 is required for kinetochore attachment and spindle integrity (Kerres et al., 2007). In the filamentous fungus *A. flavus*, we found that the Spc105 protein harbors a conserved Spc7 domain, and *spc105* gene deletion caused a delay in the nuclear division cycle (Figures 1, 4). Taken together, these results illustrate that Spc105 holds a conserved role in mitosis in various organisms. The mitotic delay caused by the loss of *spc105* may be due to the activation of a cell cycle checkpoint associated with DNA replication or spindle assembly, allowing time for repair mechanisms to resolve the detected problem.

Emerging data in both yeast and humans link the cell cycle to ribosome synthesis (Dez and Tollervey, 2004; Kugler et al., 2016). A delay in the cell cycle has been shown to cause a “slow growth signature” and reduced expression of genes associated with ribosome synthesis, while increasing the transcription of genes connected to respiratory growth (O’Duibhir et al., 2014; Kugler et al., 2016). In our study, the restricted colony growth and delayed spore germination caused by *spc105* deletion, as well as the downregulated ribosome biosynthesis-related gene expression pattern in our RNA-seq data, also support this view (Figure 2 and Supplementary Figure S6). The *Δspc105* mutant exhibited varying degrees of impact on colony growth and spore germination at different temperatures, suggesting that the effect on vegetative growth established by *spc105* deletion is dependent on temperature. Furthermore, our study also found that Spc105 regulates conidiation and sclerotia production in *A. flavus* (Figure 3). During growth in PDB submerged culture, Δ*spc105* mutants produced degenerate conidiophores (Figure 3A). This phenomenon resembles the reported abnormal conidiophore development observed under submerged cultures in the *nsdC* and *nsdD* deletion strains in *A. flavus*, both of which were found to negatively regulate conidiation (Han et al., 2001; Cary et al., 2012). The significantly increased expression level of the asexual development transcriptional activator gene *brlA* in *Δspc105* suggests a negative regulatory role of Spc105 in conidia development.

Fungal morphological differentiation is always associated with secondary metabolism. Our transcriptome analysis revealed that *spc105* deletion stimulated transcriptional changes in numerous secondary metabolite cluster genes (Supplementary Table S3), most of which were downregulated including the AF gene cluster (# 54). The loss of AF production in Δ*spc105* coincided with a markedly reduced expression of AF cluster genes. This may be due to the decreased transcripts of *aflR* and *aflS* (Figure 7 and Supplementary Table S4), encoding transcription factors that act as AF cluster activators (Yu et al., 2004). In addition to AF, the biosynthesis of kojic acid, a scavenger of free radicals (Chang et al., 2011), was also positively regulated by Spc105 (Figure 5). The production of both AF and kojic acid are postulated to relieve oxidative stress (Fountain et al., 2016a,b). In fact, several oxidative stress response-related genes were differentially expressed in the Δ*spc105* strain (Supplementary Table S5), and further studies on the relationship between Spc105 and oxidative stress response is underway.

In *A. flavus*, the global regulator of secondary metabolism LaeA is required for the transcription of the AF cluster, including the pathway activator gene *aflR* (Bok and Keller, 2004; Chang et al., 2011). Previous studies indicated that *laeA* deletion in *A. flavus* led to loss of sclerotia and AF production, as well as reduced pathogenicity, while *laeA* overexpression yielded opposite phenotypes (Kale et al., 2008; Amaike and Keller, 2009; Chang et al., 2012) similar to those displayed in the Δ*spc105* mutant in our experiments (Figure 8). The reduced *laeA* expression in the Δ*spc105* strain shown in the transcriptome data indicated a correlation between Spc105 and LaeA. We further identified the direct interaction between Spc105 and LaeA (Figure 9) and proved that the two proteins share interdependent functions in the control of AF biosynthesis (Figure 8). LaeA is a putative methyltransferase that contains a SAM domain required for its function (Bok and Keller, 2004). The primary role of LaeA is to regulate secondary metabolic gene clusters, and one of the proposed mechanisms for its regulation is that LaeA differentially methylates histone proteins and then alters chromatin structure to modulate gene expression (Palmer and Keller, 2010). However, the exact mechanism of how LaeA regulates secondary mechanisms remains enigmatic. Our findings showing that Spc105 can interact with the SAM binding domain of LaeA may provide new clues for interpreting the molecular functions of LaeA. Further studies will examine whether Spc105 correlates with chromatin remodeling and how LaeA is involved in this process.

Previous studies in *A. nidulans* showed that the light-sensitive protein VeA bridges VelB and LaeA to form the nuclear velvet complex in the dark, and is required for LaeA to control secondary metabolism (Bok and Keller, 2004; Bayram et al., 2008). Since both LaeA and Spc105 located in the nucleus while VeA shuttles between the cytoplasm and nucleus, we speculated that Spc105 may compete with VeA for binding to LaeA. Our hypothesis was supported by mapping of the interaction domains of VeA-LaeA and Spc105-LaeA (Figure 10). Additionally, it is also important to note the induced expression of the velvet family members *velB* and *velC* in the *Δspc105* background (Table 1). In *A. nidulans*, the VeA-VelB dimer functions to activate sexual development, and VelB also forms a complex with VosA to repress asexual conidiation in the dark (Bayram et al., 2008, 2010). In *A. flavus*, VelB in concert with VeA has a positive effect on conidiation (Chang et al., 2013). Thus, the transcriptional change of *velB* might be partially responsible for the observed promoted conidiation in the *Δspc105* strain. Further research is needed to investigate the relationship between Spc105 and the velvet proteins.

The regulation of fungal development and secondary metabolism requires a coordinated interplay of regulators, and the biological properties of a protein depend on its physical interaction with other molecules in the extensive and complex networks of the cell. The Spc105-LaeA identified in our study represents a communication between cell cycle progression and the velvet complex, which may improve our understanding of LaeA-involved regulatory networks. LaeA, in association with the velvet family proteins, was proposed to be involved in supporting the development of progeny in *Aspergillus* by controlling the production of secondary metabolites, which is considered beneficial for fungal survival in their ecological niche (Bayram et al., 2010; Calvo and Cary, 2015). From this perspective, we hold the opinion that LaeA functions in concert with Spc105 to improve fungal survival capability. It will be interesting to see whether this interaction is conserved in other fungi and whether LaeA can form complexes with other cell cycle related proteins.

## Data Availability

RNA-seq data were deposited in the NCBI Sequence Read Archive under accession number PRJNA498762.

## Author Contributions

Q-QZ and Z-MH designed the experiments. Q-QZ, LH, J-YL, JL, Z-LW, and G-YH performed the experiments. Q-QZ and LH analyzed the data. Q-QZ and LH wrote the manuscript. Z-MH revised the manuscript.

## Conflict of Interest Statement

The authors declare that the research was conducted in the absence of any commercial or financial relationships that could be construed as a potential conflict of interest.

## Supplementary Material

FIGURE S1 Preparation of mutant strains used in this study. **(A)** Diagram showed the construction of Δ*spc105* and *OE:spc105* strains by a homologous recombination event. **(B)** Verification of Δ*spc105* and *OE:spc105* transformants. Left: Southern blot analysis of representative transformants; Middle: PCR and RT-PCR verification of the putative *spc105* deletion and *OE:spc105* transformants. PCR performed with primer pairs *spc*/NF and *spc*/NR and OE5F and OE3R generated the product of expected size. RT-PCR were performed with primer pairs RT-spcF and RT-spcR; Right: relative gene expression levels of *spc105* in transformants were analyzed using 2^–ΔΔCT^ analysis. **(C)** Verification of eGFP-*spc* transformants. Left: Results of PCR amplification from the two putative eGFP-*spc* transformants using primer pairs OE5F and OE3R; Right: RT-PCR results of *gfp* gene expression in two putative transformants; **(D)** Verification of *spc*/*laeA* double mutants by PCR and qRT-PCR. Left: PCR analysis of Δ*spc*-*OElaeA* transformants using primers PTF and PTR confirming pPTRI vector insertion. **(E)** Verification of *Δspc105*-*C* strain. Left: PCR analysis of *Δspc105*-*C* transformants using primers CMspc5F and CMspc partB R generated the product of expected size. Right: RT-PCR analysis of *Δspc105*-*C* strain using primer pairs RT-spcF and RT-spcR.

FIGURE S2 Hyphae dry weight measurement of *spc105* mutant strains in PDB culture. Strain spores were inoculated in 30 ml PDB broth and incubated with shaking at 200 rpm. Mycelia were harvested at indicated time points by vacuum filtration.

FIGURE S3 Aflatoxin production and peanuts colonization analysis of WT and Δ*spc105-C* strains. **(A)** Strains were cultured in 30 ml PDB culture for 48 h at 30°C, and AFB_1_ was extracted from the culture. **(B)** Photographs of peanut seeds infected with *A. flavus* strains after 3 days of incubation at 30°C.

FIGURE S4 Overlaid chromatograms analysis of several secondary metabolite production in *A. flavus spc105* mutant strains. **(A–C)** represents AFB_1_ kojic acid, and CPA, respectively. Results confirmed that little to no AFB_1_ and extreme low level AFB_1_ is present in extracts of the Δ*spc105* strain at 25 and 30°C, respectively.

FIGURE S5 Global gene expression analysis of two samples with three replications. **(A)** Cluster analysis of sample to sample to verify the similarities between parallel samples. **(B)** The boxplot of overall gene expression level of two samples with three replicates. **(C)** A volcano plot shows the total differentially expressed genes (DEGs) (red spots, *P* ≤ 0.01) between Δ*spc105* and WT samples. **(D)** The heatmap showed the DEGs between Δ*spc105* and WT samples. The data used to construct the heatmap was based on the log2 value of the FPKM values of all DEGs.

FIGURE S6 KEGG analysis of DEGs between Δ*spc105* and WT.

TABLE S1 *Aspergillus flavus* strains used in this research and their genotypes.

TABLE S2 Primers used in this study.

TABLE S3 Differentially expressed backbone genes in Δ*spc105* relative to WT. Genes with | log2 (fold change)| ≥ 1.5 and adjusted *P*-value (padj) ≤ 0.01 were defined as significantly differentially expressed.

TABLE S4 Transcript levels relative to WT for AF cluster genes in Δ*spc105*.

TABLE S5 Differentially expressed oxidative stress response genes in *Δspc105* relative to WT.
