# Supplementary figures and images for: The Kinetochore Protein Spc105, a Novel Interaction Partner of LaeA, Regulates Development and Secondary Metabolism in Aspergillus flavus
Source: Front Microbiol. 2019 Aug 13;10:1881. doi: 10.3389/fmicb.2019.01881 (PMC6700525; doi:10.3389/fmicb.2019.01881)

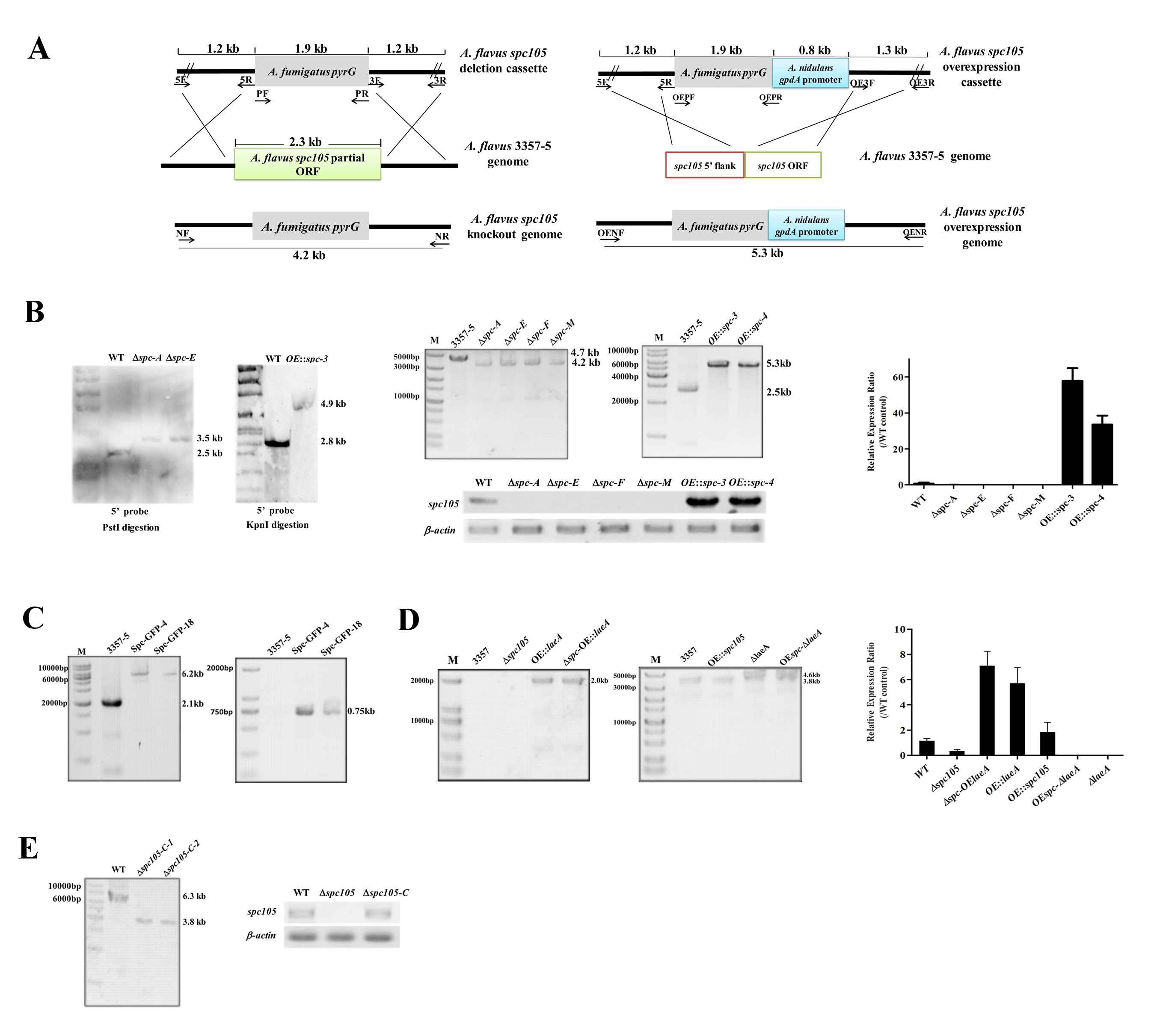

Supplement: FIGURE S1 — Preparation of mutant strains used in this study. (A) Diagram showed the construction of Δspc105 and OE:spc105 strains by a homologous recombination event. (B) Verification of Δspc105 and OE:spc105 transformants. Left: Southern blot analysis of representative transformants; Middle: PCR and RT-PCR verification of the putative spc105 deletion and OE:spc105 transformants. PCR performed with primer pairs spc/NF and spc/NR and OE5F and OE3R generated the product of expected size. RT-PCR were performed with primer pairs RT-spcF and RT-spcR; Right: relative gene expression levels of spc105 in transformants were analyzed using 2–ΔΔCT analysis. (C) Verification of eGFP-spc transformants. Left: Results of PCR amplification from the two putative eGFP-spc transformants using primer pairs OE5F and OE3R; Right: RT-PCR results of gfp gene expression in two putative transformants; (D) Verification of spc/laeA double mutants by PCR and qRT-PCR. Left: PCR analysis of Δspc-OElaeA transformants using primers PTF and PTR confirming pPTRI vector insertion. (E) Verification of Δspc105-C strain. Left: PCR analysis of Δspc105-C transformants using primers CMspc5F and CMspc partB R generated the product of expected size. Right: RT-PCR analysis of Δspc105-C strain using primer pairs RT-spcF and RT-spcR. [file Image_1.TIF]

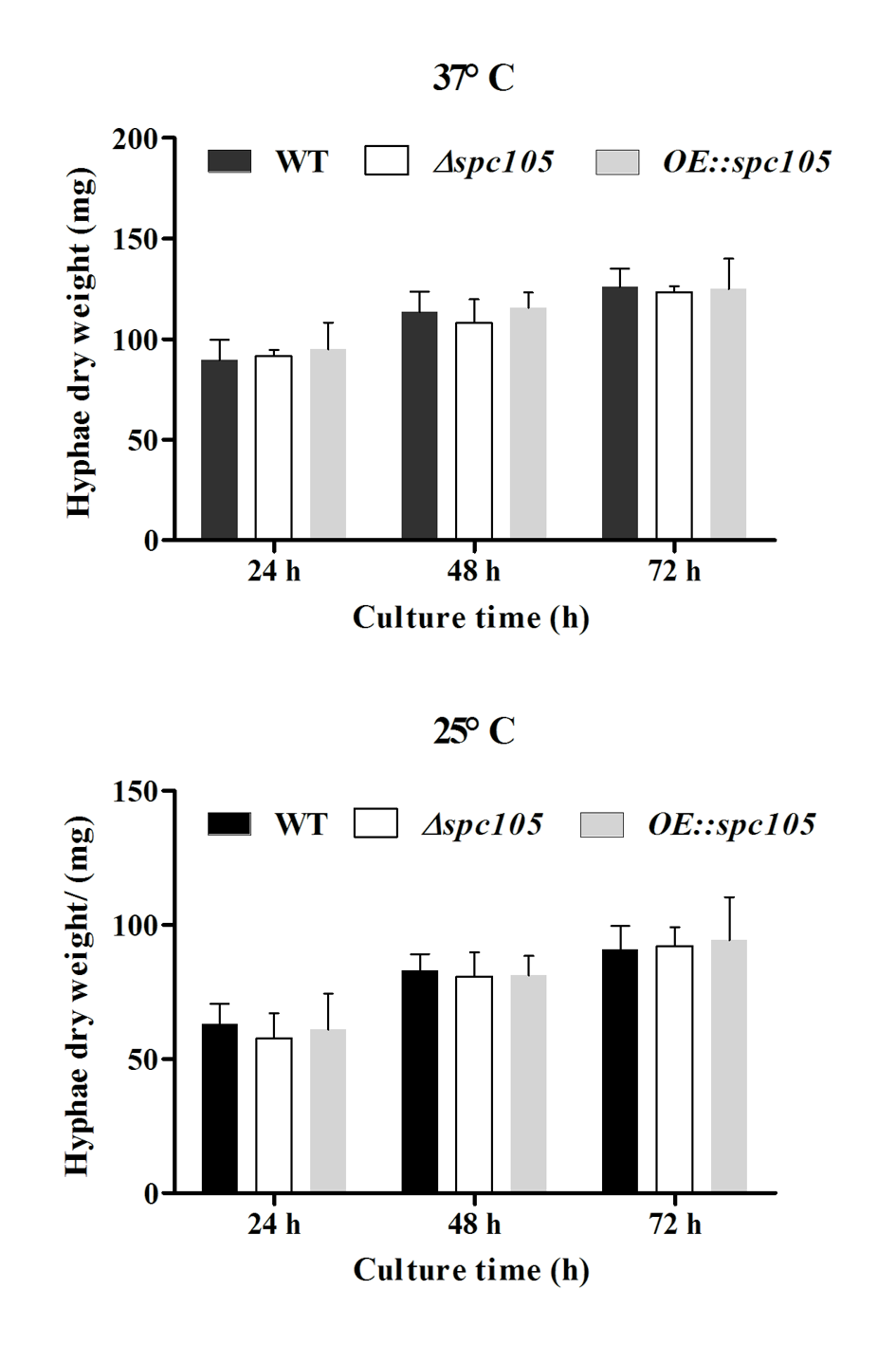

Supplement: FIGURE S2 — Hyphae dry weight measurement of spc105 mutant strains in PDB culture. Strain spores were inoculated in 30 ml PDB broth and incubated with shaking at 200 rpm. Mycelia were harvested at indicated time points by vacuum filtration. [file Image_2.TIF]

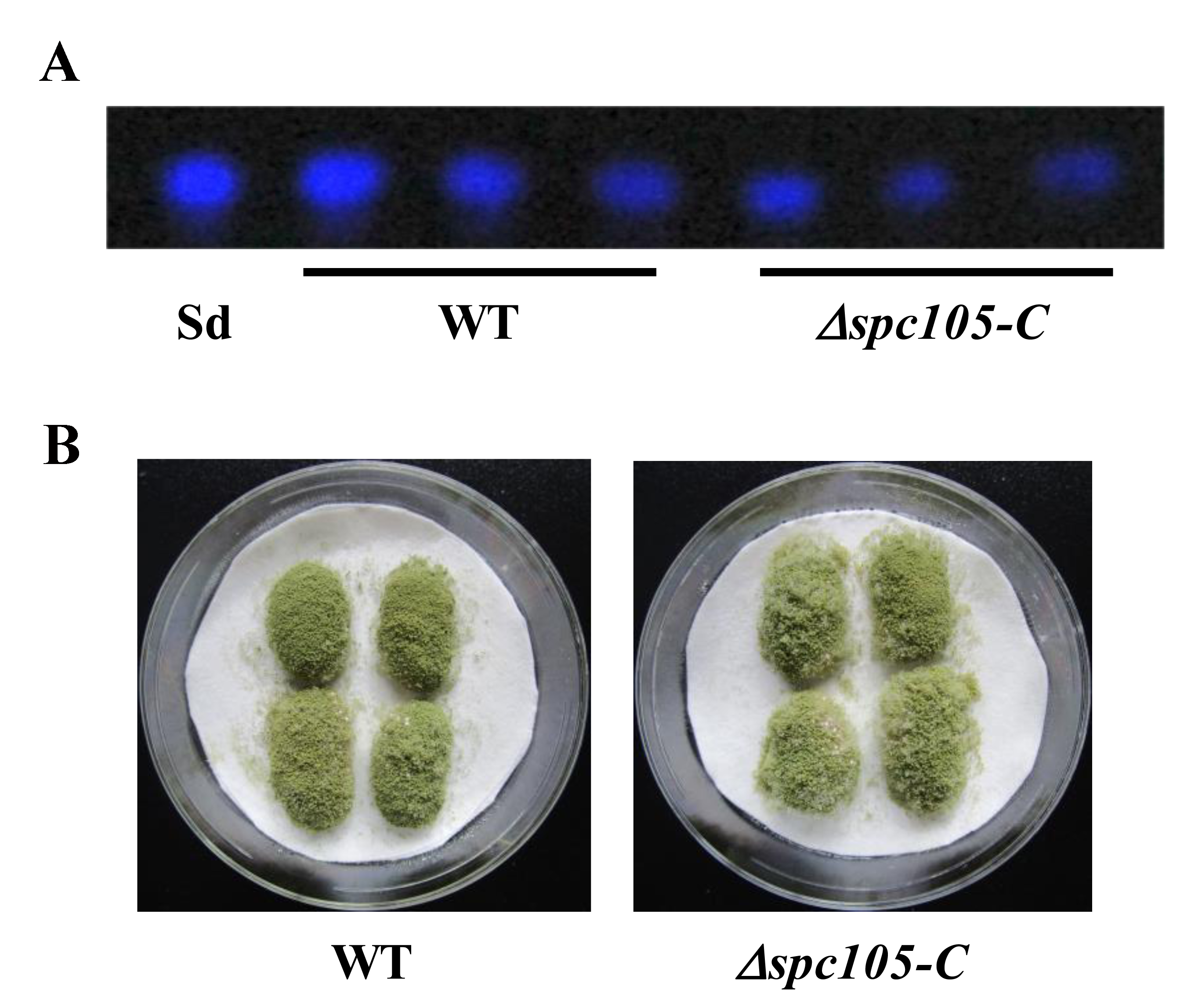

Supplement: FIGURE S3 — Aflatoxin production and peanuts colonization analysis of WT and Δspc105-C strains. (A) Strains were cultured in 30 ml PDB culture for 48 h at 30°C, and AFB1 was extracted from the culture. (B) Photographs of peanut seeds infected with A. flavus strains after 3 days of incubation at 30°C. [file Image_3.TIF]

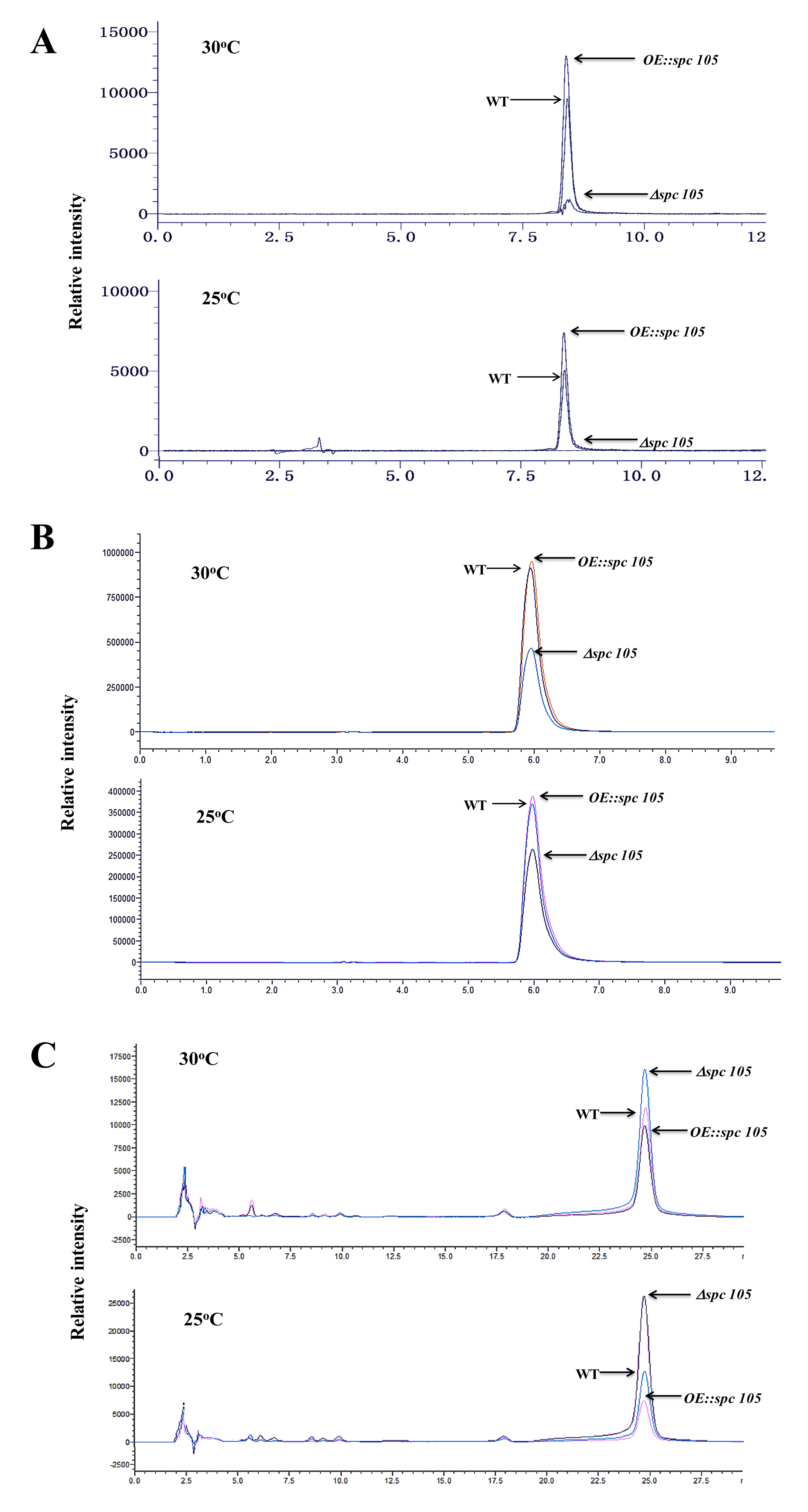

Supplement: FIGURE S4 — Overlaid chromatograms analysis of several secondary metabolite production in A. flavus spc105 mutant strains. (A–C) represents AFB1 kojic acid, and CPA, respectively. Results confirmed that little to no AFB1 and extreme low level AFB1 is present in extracts of the Δspc105 strain at 25 and 30°C, respectively. [file Image_4.TIF]

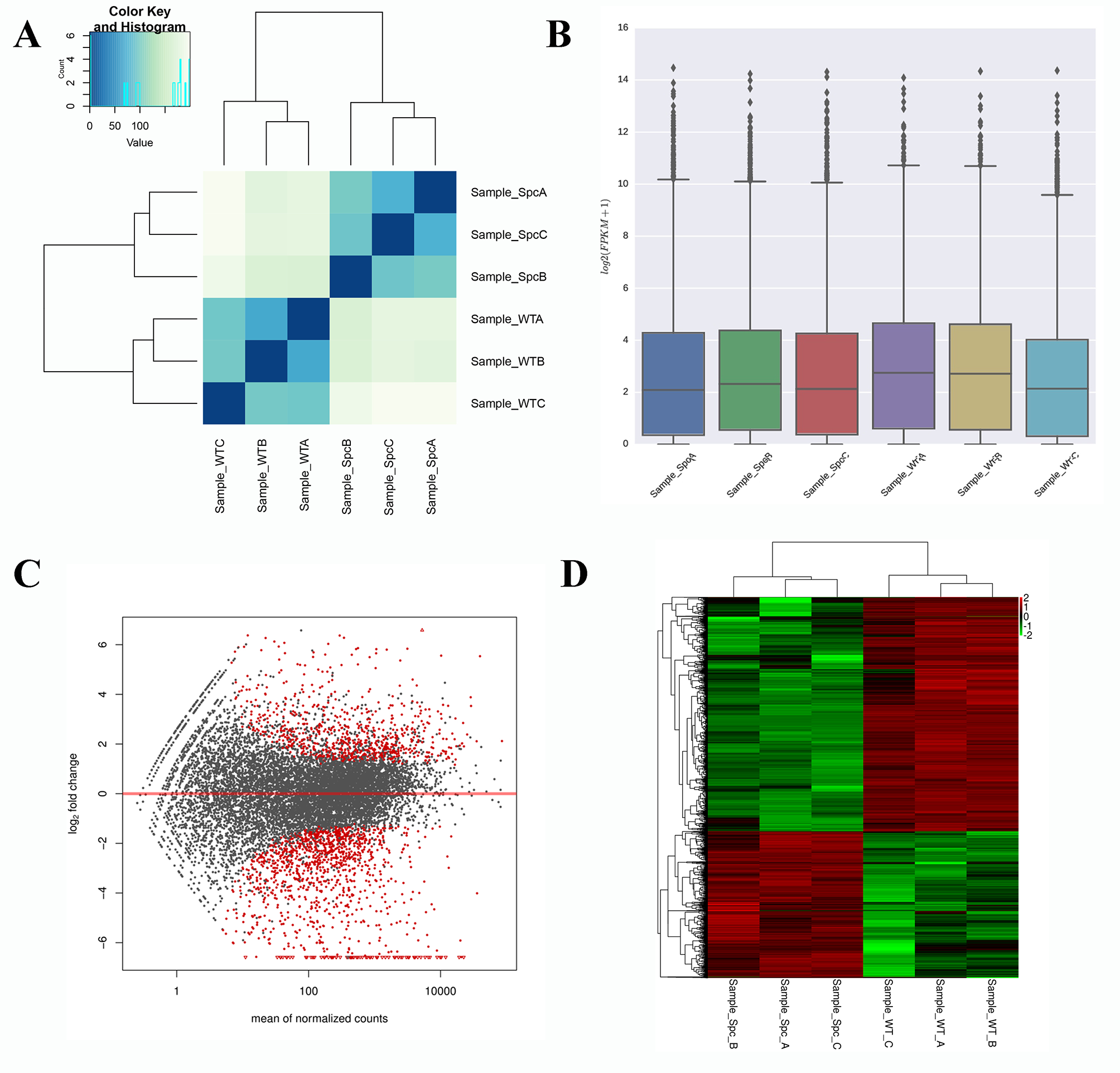

Supplement: FIGURE S5 — Global gene expression analysis of two samples with three replications. (A) Cluster analysis of sample to sample to verify the similarities between parallel samples. (B) The boxplot of overall gene expression level of two samples with three replicates. (C) A volcano plot shows the total differentially expressed genes (DEGs) (red spots, P ≤ 0.01) between Δspc105 and WT samples. (D) The heatmap showed the DEGs between Δspc105 and WT samples. The data used to construct the heatmap was based on the log2 value of the FPKM values of all DEGs. [file Image_5.TIF]

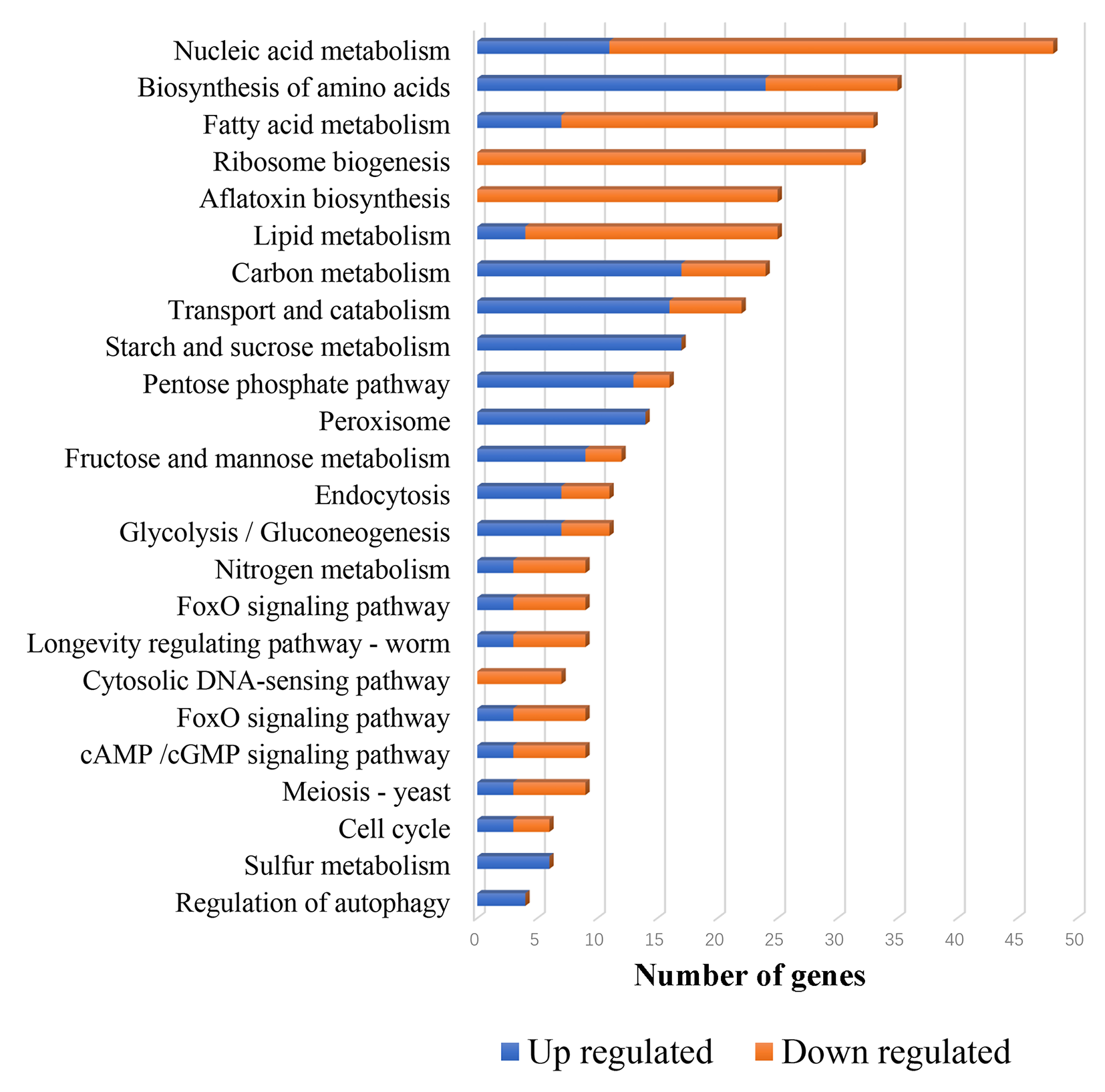

Supplement: FIGURE S6 — KEGG analysis of DEGs between Δspc105 and WT. [file Image_6.TIF]
