# Supplementary material for: The Kinetochore Protein Spc105, a Novel Interaction Partner of LaeA, Regulates Development and Secondary Metabolism in Aspergillus flavus
Source: Front Microbiol. 2019 Aug 13;10:1881. doi: 10.3389/fmicb.2019.01881 (PMC6700525; doi:10.3389/fmicb.2019.01881)
Supplement: TABLE S1 — Aspergillus flavus strains used in this research and their genotypes. [file Table_1.docx]

| **Table S1. *Aspergillus ﬂavus* strains used in this research and their genotypes** | | |
| --- | --- | --- |
| **Strain** | **Genotype** | **Strain source** |
| NRRL3357 | Wild type | (Payne et al., 1993) |
| NRRL3357-5 | *pyrG*^―^ | (He, et al., 2007) |
| *∆spc105* | ∆*spc105*::*A. fumigatus pyrG*, *pyrG*^―^ | This study |
| *OE::spc105*  *∆spc105-C* | *A. fumigatus pyrG*::*gpdAp*::*spc105*, *pyrG*^―^  ∆*spc105*::*A. fumigatus pyrG*, *Aspergillus oryzae* *ptrA*::*spc105*, *pyrG*^―^ | This study  This study |
| Spc-GFP | *A. fumigatus pyrG*::*gpdAp*::*eGFP*::*spc105*, *pyrG*^―^ | This study |
| *∆spc-OElaeA* | ∆*spc105*::*A. fumigatus pyrG*, *Aspergillus oryzae* *ptrA*::*gpdAp*::*laeA, pyrG*^―^ | This study |
| *OE::laeA* | *A. oryzae* *ptrA*::*gpdAp*::*laeA* | This study |
| *OEspc-∆laeA* | *A. fumigatus pyrG*::*gpdAp*::*spc105*, ∆*laeA*::*A. oryzae* *ptrA*, *pyrG*^―^ | This study |
| *∆laeA*  TXZ21.3  LH-1  LS-1 | ∆*laeA*::*A. oryzae* *ptrA*  ∆*ku70,* ∆*argB, pyrG*^―^  *A. flavus* *argB::gpdAp::laeA::HA*, ∆*ku70, pyrG*^―^  *A. flavus* *argB::gpdAp::laeA::HA*, *A. fumigatus pyrG*::*gpdAp*::3xflag::*spc105*, ∆*ku70* | This study  (Zhao et al., 2016)  This study  This study |
