# Supplementary material for: The Kinetochore Protein Spc105, a Novel Interaction Partner of LaeA, Regulates Development and Secondary Metabolism in Aspergillus flavus
Source: Front Microbiol. 2019 Aug 13;10:1881. doi: 10.3389/fmicb.2019.01881 (PMC6700525; doi:10.3389/fmicb.2019.01881)
Supplement: TABLE S2 — Primers used in this study. [file Table_2.docx]

| **Table S2 Primers used in this study** | | |
| --- | --- | --- |
| **Name** | **Sequence (5' to 3')** | **Purpose** |
| spc/5F | GCTTTCCAACCCTGGATTAAG | *A. flavus spc105* 5' flank |
| spc/5R | GAAGAGCATTGTTTGAGGCATTAAGCAGCTACCTTGGGTGAC |  |
| pyrG/F | GTCACCCAAGGTAGCTGCTTAATGCCTCAAACAATGCTCTTC | *A. fumigatus* *pyrG* |
| pyrG/R | ATATCCCGTTGCTCATAACCAGGTATCGTCGGGAGGT |  |
| spc/3F | ACCTCCCGACGATACCTGGTTATGAGCAACGGGATAT | *A. flavus spc105* 3' flank |
| spc/3R | GAATTGACGATAACTAATAC |  |
| spc/NF | AAAGCACCATCCGTCGCAAG | *A. flavus spc105* deletion product |
| spc/NR | CCGCCGCCAAAAGTAAGCAC |  |
| OE5F | GTACGGGGCAGGACACGGAG | *A. flavus* OE*spc105* 5' flank |
| OE5R | GTGAAGAGCATTGTTTGAGGCAAAGGCAAGATATAGAGTATC |  |
| OEpyrG/F | CTATGATACTCTATATCTTGCCTTTGCCTCAAACAATGCTCTTC | *A. fumigatus* *pyrG* for OE*spc105* |
| OEpyrG/R | CAAGCCTTCGACATCCGGATGCCAGGTATCGTCGGGAGGT |  |
| gpdA/F | ACCTCCCGACGATACCTGGCATCCGGATGTCGAAGGCTTG | *A. nidulans gpdA* promoter |
| gpdA/R | GAGTCACTTCTGGATGCCATCATTGTGATGTCTGCTCAAG |  |
| OE3F | CTTGAGCAGACATCACAATGATGGCATCCAGAAGTGACTC | *A. flavus* OE*spc105* 3' flank |
| OE3R | CCATTGACATCTCGCCGTTC |  |
| OENF | GAGTTTCAAGAACGATTGTG | *A. flavus spc105* OE product |
| OENR | CGTCGTCTAAGCTCATTGCAGT |  |
| CMspc5F  CMspc5R  CMspc partB F  CMspc partB R | GAAACAGCTATGACCATGATTACGCCATGGCAATCCCCTTTCCCGG  GCCAATAGACCGGCGCTTATCAAGCATAAGGCAAGATATAGAGTATC  GATACTCTATATCTTGCCTTATGCTTGATAAGCGCCGGTCTATTGGC  GACGGCCAGTGAATTCGAGCTCGGTACCTTAAAACACTGTCCCGGTAAG | *spc105* deletion complementation vector construction |
| GFP/P1 | AGTATATTCATCTTCCCATGGTGAGCAAGGGCGAGGAG | GFP fragment |
| GFP/P2 | CGCGGAGTCACTTCTGGATGCCATCTTGTACAGCTCGTCCATGC |  |
| GL/F | CCCAAGCTTCCGGTGACTCTTTCTGGC | *A. nidulans gpdA* promoter |
| GL/R | CTTTGCGGTACGCATGATACATGGGAAGATGAATATACTG |  |
| LG/F | CAGTATATTCATCTTCCCATGTATCATGCGTACCGCAAAG | *A. flavus* *laeA* ORF |
| LG/R | GGGGTACCTCAGTTCGCAGGTTTCCGTG |  |
| laeA/5F | CTCAGGCTTCTTCCTTAACCG | *A. flavus laeA* 5' flank |
| laeA/5R | GATCCCGTAATCAATTGCCCTCGTCCGTTTTCTTCATAGA |  |
| ptrA/F | TCTATGAAGAAACGGACGAGGGCAATTGATTACGGGATC | *A. nidulans* *ptrA* gene |
| ptrA/R | GCGCATGTTGGTAGAAGAAATGGGGTGACGATGAGCCGC |  |
| laeA/3F | GCGGCTCATCGTCACCCCATTTCTTCTACCAACATGCGC | *A. flavus laeA* 3' flank |
| laeA/3R | CCTGCCGAAGAAATTCACC |  |
| laeA/NF | GCTTTCCATTACAGGGCTTC | *A. flavus laeA* deletion product |
| laeA/NR | GGCGAAATACCCAAAGACT |  |
| AD-spcF (NdeI)  AD-spcR (EcoRI) | gagtacccatacgacgtaccagattacgctATGGCATCCAGAAGTGACTC atggatcccgtatcgatgcccacccgggtgTTAAAACACTGTCCCGGTAA | Y2H AD-spc full length |
| BD-spcF (NdeI)  BD-spcR (EcoRI) | gagcagaagctgatctcagaggaggacctgATGGCATCCAGAAGTGACTC  tgcggccgctgcaggtcgacggatccccggTTAAAACACTGTCCCGGTAA | Y2H BD-spc full length |
| AD-spc (1-1004) R | atggatcccgtatcgatgcccacccgggtgTGGCCCGGATTCTTCCTG | Y2H spc truncation |
| AD-spc (1-1328) R | atggatcccgtatcgatgcccacccgggtgGGCCCCCGGGTGGAACGAAAG |  |
| AD-spc (1005-1328) F | gagtacccatacgacgtaccagattacgctGAAGTTGAGCCTATCCAATTG |  |
| AD-spc (1329-1541) F | gagtacccatacgacgtaccagattacgctTTCTTTATTGATAATAGTAAC |  |
| AD-spc (1005-1168) R | GGCCCAAGTATTCTGCTTTAAGTTCATCATCACCTCTCA |  |
| AD-spc (1253-1328) F | TGAGAGGTGATGATGAACTTAAAGCAGAATACTTGGGCC |  |
| AD-laeA F (NdeI)  AD-laeA R (EcoRI) | gagtacccatacgacgtaccagattacgctATGTATCATGCGTACCGC  atggatcccgtatcgatgcccacccgggtgTCAGTTCGCAGGTTTCCGTG | Y2H laeA truncation |
| BD-laeA F (NdeI)  BD-laeA R (EcoRI) | gagcagaagctgatctcagaggaggacctgATGTTTGGAAACGGCCAG  tgcggccgctgcaggtcgacggatccccggTCAGTTCGCAGGTTTCCGTG |  |
| BD-laeA (1-127) R | tgcggccgctgcaggtcgacggatccccggGTACATTAGGCCATCCGAC |  |
| BD-laeA (128-285) F  BD-laeA (128-285) R | gagcagaagctgatctcagaggaggacctgGCCCCGCATCCCAGGAAC  tgcggccgctgcaggtcgacggatccccggAATATCGGTAAAACCAGC |  |
| BD-laeA (286-369) F | gagcagaagctgatctcagaggaggacctgGATCATCAAATGGTGGGGTTG |  |
| AD-veA F  AD-veA R | gagtacccatacgacgtaccagattacgct ATGGCGACACGAGCTCCTTTG  atggatcccgtatcgatgcccacccgggtg TTACTGGAGTGCAGGGGATATC | Y2H veA |
| BD-veA F  BD-veA R | gagcagaagctgatctcagaggaggacctg ATGGCGACACGAGCTCCTTTG  tgcggccgctgcaggtcgacggatccccgg TTACTGGAGTGCAGGGGATATC |  |
| AD-velB F  AD-velB R | gagtacccatacgacgtaccagattacgctATGTATGCTATCGAAGAAAG  atggatcccgtatcgatgcccacccgggtgTTAATCGTAATCGTCCCCATC | Y2H velB |
| BD-velB F  BD-velB R | gagcagaagctgatctcagaggaggacctgATGTATGCTATCGAAGAAAG  tgcggccgctgcaggtcgacggatccccggTTAATCGTAATCGTCCCCATC |  |
| LaeA-pET28a F (BamHI)  LaeA-pET28a R (SalI) | catatgactggtggacagcaaatgggtcgcATGTATCATGCGTACCGC  tggtggtgctcgagtgcggccgcaagcttgTCAGTTCGCAGGTTTCCGTG | GST pull-down |
| Spc-pGEX4T-1F (EcoRI)  Spc-pGEX4T-1R (NotI) | ctggttccgcgtggatccccggaaATGCTTGATAAGCGCCGGT  accggcgcttatcaagcatTTAAAACACTGTCCCGGTAA |  |
| laeA5’flank R  argBF  argBR  GpdA F( LH1)  GpdA R( LH1)  laeA F(LH1)  laeA F(LH1)  laeA3’flank F  laeA3’flank R | TCGTCCGTTTTCTTCATAGAAAGGCTGTTTTTCAGAACTCCT  AGGAGTTCTGAAAAACAGCCTTTCTATGAAGAAAACGGACGA  CTCCGCATGCCAGAAAGAGTCACCGGCCCGTGACATGTGAATGCG  GTGCCCGCATTCACATGTCACGGGCCGGTGACTCTTTCTGGC  GTCCAGTCTGGCCGTTTCCAAACATGGGAAGATGAATATACTGAAG  CCATCTTCAGTATATTCATCTTCCCATGTTTGGAAACGGCCAGAC  AGCGTAGTCTGGGACGTCGTATGGGTAGTTCGCAGGTTTCCGTGCTTG  TACCCATACGACGTCCCAGACTACGCTTGATTCTTCTACCAACATGC  TGTATGAGTTCACATTATAG | C-terminal tagged LaeA |
| GpdA R(3xflag)  SpcF (3xflag) | CTTGTAATCGATCTTATCGTCGTCATCCTTGTAATCTCCCTTATCGTCGTCATCCTTGTAATCCATGGGAAGATGAATATACTG  TAAGGGAGATTACAAGGATGACGACGATAAGATCGATTACAAGGATGACGACGATAAGATGGCATCCAGAAGTGACTCC | N-terminal tagged Spc105 |
| RT-spcF  RT-spcR | ATGCTTGATAAGCGCCGGT  TTAAAACACTGTCCCGGTAA | RT-PCR |
| spc-F  spc-R | GCTTTATCAACATTCTTGCCGAG  TTCATCCGCCACTCATACCAA | QPCR |
| laeA-F  laeA-R | ATCTCCTCCGCCCAATACCCA  TCATATCGGCCTCTGTGCTTT |  |
| veA-F  veA-R | ACGACGACCTCAACAAGACC  AAGTAAGCCACTCCAGCCAC |  |
| velB-F  velB-R | TGAGCTTTGTCAACGTGGGT  AAATGGCTCGGAGAAGACGG |  |
| brlA-F  brlA-R | TATCCAGACATTCAAGACGCACAG  GATAATAGAGGGCAAGTTCTCCAAAG |  |
| wetA-F  wetA-R | AGCATTTTCACCCCATCTCCC  TTGATAACACTCCCGTACATGGC |  |
| abaA-F  abaA-R | GAGTGGCAGACCGAATGTATGTTG  TAGTGGTAGGCATTGGGTGAGTTG |  |
| actin-F  actin-R | AGGACTCTTATGTCGGTGATG  CGGTTGGACTTAGGGTTG |  |
| aflA-F  aflA-R | CCTCCCTCTGATGCCGGTGA  ATTTCAATGCAGCGACCCGAT |  |
| aflB-F  aflB-R | TCAAGCCCGTACCATTCATCC  ATTACCGCCTCCACATCTTCG |  |
| aflC-F  aflC-R | TGTCAGACCACAAACGCACCT  CATCTCACAGAACGCCCTCAA |  |
| aflD-F  aflD-R | CGCCTGAGGAGACGGTGTATT  CTGCCTTCAGCGACGGTTAG |  |
| aflE-F  aflE-R | ACAGAAGAACACCAAGGCTAC  CGGATTTCCTCCTCGCTCAG |  |
| aflF-F  aflF-R | TCCTGGATTTCCGCATACCT  ATTGGCTTCGCTCCCTCTAC |  |
| aflG-F  aflG-R | GACTCGTTCGGCTGCCTTGA  GTTCCGGCGTCGCTGTTCTA |  |
| aflH-F  aflH-R | CACCCCACTGATTGCGCCTA  ATCGCCTCCCCAACCATACTCG |  |
| aflI-F  aflI-R | CTTCGCATTCAAATCCTCGTT  CGCACATGAACACAATACTAGCA |  |
| aflJ-F  aflJ-R | CTGCGTTGCTACACTCCCC  ATCACGCGGCAGAAACCATC |  |
| aflK-F  aflK-R | CCTCAACATCTTTACACGCACT  CCCGATTCCAGACACCATTAGCA |  |
| aflL-F  aflL-R | CCGACAAGTTCATCCCCGAG  TGCGCCCAATACAGTTCCG |  |
| aflM-F  aflM-R | CCGTTTAGATGGCAAAGTGGC  TCACGGGAATGGGCGTAGTT |  |
| aflN-F  aflN-R | ACCTTCCTATGCCAGAGTTCA  AATCATCCAGGTCCCGCTA |  |
| aflO-F  aflO-R | TTCGACATCTCGGGACCTTGC  GGGACGCTCCAGAGCCATTAA |  |
| aflP-F  aflP-R | TCTAACATCGTCACCGCCAT  GCCACCATATCTTCTCAGTCTCC |  |
| aflQ-F  aflQ-R | TAAGGCAGCGGAATACAAGCG  CAAGAGGATGGAAGGACGAGA |  |
| aflR-F  aflR-R | GCAGTCAATGGAACACGGAAAC  CCTGAAACGGTGGTAGTGGG |  |
| aflS-F  aflS-R | CTGACCATCTCCGACCCGTTC  AGAGCCAACTGTCGGACCAA |  |
| aflT-F  aflT-R | AGTAGTCACATCTCCGGCAT  CAGGATGGTGTTATCGAGAG |  |
| aflU-F  aflU-R | CCTCGGCATCAATTTCGCTCT  CAATCCGCCAACCCAGTCACC |  |
| aflV-F  aflV-R | AGACCTGCCTATCCTGAATGCC  CAAATGTATGAGGGTCTTTC |  |
| aflW-F  aflW-R | TGGCACATACTACCGTGTCG  GTTGTCGTCGCATCACCCC |  |
| aflX-F  aflX-R | CACACTCTCAGCACAACCAC  GCCAGTCATCACATAGTACACC |  |
| aflY-F  aflY-R | GATTTCACAACCACATTAGCC  TGTTAAAGTCATACGCAGCTTG |  |
| kojA-F  kojA-R | TAGTCGTCCACCATGAATCCG  TCATCCAGATTGTATGCGCTGA |  |
| kojR-F  kojR-R | GCGCTATTTTCCTGTTCGTT  AATTGTTTTGCCTTCCACTGC |  |
| kojT-F  kojT-R | AGTTGTTCCACCATGCACA  CCACCCAGAATAACCGTCT |  |
| cdc7F  cdc7R | ACATTAAACCGACCAATTTCC  ACCAAAACACCTTCTCGGAT |  |
| cdkF  cdkR | TCGGAGAAGGGACTTATGG  TTCATCGTCTGTCTCTAATCGG |  |
| mcm2F  mcm2R | GCAGACATGCGTCGTGAATC  GGAAACCTTCTGACTGCCGA |  |
| mcm3F  mcm3R | TGTGTGGCCTTGTAAAAGTG  CGGTTATGTGCTCTGATCTC |  |
| mcm4F  mcm4R | GACATCCGCTCCTCTGATCG  ACTGCCTCCTCAACATCTGC |  |
| mcm5F  mcm5R | TGAAGCGCAGACTTCCCATT  TACTCCGTGACGGTACACCT |  |
| mcm6F  mcm6R | GCGAATGGCGGTGGTATTTG  CAACTTGGTCGCTGATGTCC |  |
| mcm7F  mcm7R | CTGCATCCGTCTCGGACTAC  GTGAAAGACGAACAACGCCC |  |
| nimE-F  nimE-R | GAGCGTGGGCCTTGGGATG  AGACAAGAGATGTGCTTACCTT |  |
| nimO-F  nimO-R | CCGGGCTACAGATGCTACAG  GTTTCGCCGTTTTCCAGCAT |  |
