# Supplementary material for: The Kinetochore Protein Spc105, a Novel Interaction Partner of LaeA, Regulates Development and Secondary Metabolism in Aspergillus flavus
Source: Front Microbiol. 2019 Aug 13;10:1881. doi: 10.3389/fmicb.2019.01881 (PMC6700525; doi:10.3389/fmicb.2019.01881)
Supplement: TABLE S3 — Differentially expressed backbone genes in Δspc105 relative to WT. Genes with | log2 (fold change)| ≥ 1.5 and adjusted P-value (padj) ≤ 0.01 were defined as significantly differentially expressed. [file Table_3.DOCX]

| **Table S3 Differentially expressed backbone genes in *Δspc105* relative to WT (*p* < 0.01)** | | | |
| --- | --- | --- | --- |
|  | **Accession** | **Description** | **Cluster** |
| **Up-regulated** | AFLA_002900 | polyketide synthase, putative | 1 |
|  | AFLA_008770 | nonribosomal peptide synthase, putative | 6 |
|  | AFLA_010010 | nonribosomal peptide synthase, putative | 8 |
|  | AFLA_070920 | NRPS-like enzyme, putative | 25 |
|  | AFLA_105450 | polyketide synthase, putative | 38 |
|  | AFLA_126710 | polyketide synthase, putative | 50 |
| **Down-regulated** | AFLA_009120 | NRPS-like enzyme, putative | 7 |
|  | AFLA_010620 | Nonribosomal siderophore peptide synthase Sid2 | 9 |
|  | AFLA_016140 | conidial pigment biosynthesis scytalone dehydratase Arp1 | 10 |
|  | AFLA_023020 | NRPS-like enzyme, putative | 11 |
|  | AFLA_047190 | L-ornithine N5-oxygenase SidA | 16 |
|  | AFLA_047200 | palmitoyltransferase SidR | 16 |
|  | AFLA_060680 | dimethylallyl tryptophan synthase, putative | 19 |
|  | AFLA_069330 | nonribosomal peptide synthase Pes1 | 24 |
|  | AFLA_079380 | NRPS-like enzyme, putative | 26 |
|  | AFLA_104210 | PKS-like enzyme, putative | 36 |
|  | AFLA_104250 | PKS-like enzyme, putative | 36 |
|  | AFLA_116220 | polyketide synthase, putative | 42 |
|  | AFLA_118440 | NRPS-like enzyme, putative | 45 |
|  | AFLA_126710 | polyketide synthase, putative | 50 |
|  | AFLA_139410 | *aflC* / *pksA* / *pksL1* / polyketide synthase | 54 |
|  | AFLA_139480 | Dimethylallyl tryptophan synthase, putative | 55 |
|  | AFLA_139490 | Hybrid polyketide synthase–nonribosomal peptide synthetase enzyme | 55 |
|  | AFLA_096050 | protein RDR1, putative | 56 |
