# Supplementary material for: The Kinetochore Protein Spc105, a Novel Interaction Partner of LaeA, Regulates Development and Secondary Metabolism in Aspergillus flavus
Source: Front Microbiol. 2019 Aug 13;10:1881. doi: 10.3389/fmicb.2019.01881 (PMC6700525; doi:10.3389/fmicb.2019.01881)
Supplement: TABLE S4 — Transcript levels relative to WT for AF cluster genes in Δspc105. [file Table_4.DOCX]

| **Table S4. Transcript levels relative to WT for AF cluster genes in *Δspc105*** | | | | |
| --- | --- | --- | --- | --- |
|  |  | **RNA-Seq^a^** | **qRT-PCR^b^** | |
| **Locus tag** | **Gene name** | ***Δspc105*** | ***Δspc105*** | ***OE::spc105*** |
| AFLA_139100 | *aflYe* | 1.3450 | 0.9049 | 0.7993 |
| AFLA_139110 | *aflYd* | 1.6403 | 1.0649 | 0.9796 |
| AFLA_139120 | *aflYc* | 0.0568 | 0.4202 | 0.8756 |
| AFLA_139130 | *aflYb* | 0.6171 | 1.1021 | 1.3021 |
| AFLA_139140 | *aflYa* | 0.0051 | 0.3233 | 0.9844 |
| AFLA_139150 | *aflY* | 0.0091 | 0.1919 | 0.5364 |
| AFLA_139160 | *aflX* | 0.0087  0.0034  0.0044  0.0040  0.0020 | 0.0220 | 1.0945 |
| AFLA_139170 | *aflW* | 0.0034 | 0.0419 | 1.0145 |
| AFLA_139180 | *aflV* | 0.0044 | 0.0133 | 0.4526 |
| AFLA_139190 | *aflK* | 0.0040 | 0.0156 | 3.03433 |
| AFLA_139200 | *aflQ* | 0.0020 | 0.0032 | 0.7571 |
| AFLA_139210 | *aflP* | 0.0018 | 0.0053 | 0.6801 |
| AFLA_139220 | *aflQ* | 0.0024 | 0.0073 | 3.4365 |
| AFLA_139230 | *aflI* | 0.0036 | 0.0912 | 5.3455 |
| AFLA_139240 | *aflLa* | 0.0033 | 0.0533 | 0.8904 |
| AFLA_139250 | *aflL* | 0.0050 | 0.0084 | 0.6544 |
| AFLA_139260 | *aflG* | 0.0036 | 0.0062 | 4.7922 |
| AFLA_139270 | *aflNa* | 0.0068 | 0.1704 | 1.5677 |
| AFLA_139280 | *aflN* | 0.0272 | 0.0427 | 2.3567 |
| AFLA_139290 | *aflMa* | 0.0009 | 0.0014 | 1.0995 |
| AFLA_139300 | *aflM* | 0.0089 | 0.0365 | 6.7983 |
| AFLA_139310 | *aflE* | 0.0125 | 0.1803 | 0.9502 |
| AFLA_139320 | *aflJ* | 0.0233 | 0.0956 | 4.7954 |
| AFLA_139330 | *aflH* | 0.0194 | 0.1094 | 0.5032 |
| AFLA_139340 | *aflS* | 0.3059 | 0.3904 | 2.1983 |
| AFLA_139360 | *aflR* | 0.0550 | 0.1355 | 1.2781 |
| AFLA_139370 | *aflB* | 0.0366 | 0.0765 | 3.5865 |
| AFLA_139380 | *aflA* | 0.0166 | 0.0233 | 7.4697 |
| AFLA_139390 | *aflD* | 0.0058 | 0.0435 | 1.5602 |
| AFLA_139400 | *aflCa* | 0.0015 | 0.1081 | 0.9875 |
| AFLA_139410 | *aflC* | 0.0166 | 0.0398 | 2.9709 |
| AFLA_139420 | *aflT* | 0.0069 | 0.0973 | 1.0981 |
| AFLA_139430 | *aflU* | 0.0231 | 0.0353 | 1.9893 |
| AFLA_139440 | *aflF* | 0.0422 | 0.0892 | 3.1455 |
| **a Relative expression from RNA-seq data of *Δspc105* strain.**  **b Values are expression levels of *Δspc105* strain and *OE::spc105* strain relative to those of WT from qRT-PCR. All analyses were performed in biological triplicate. The standard deviations of the triplicates are within 10%.** | | | | |
