# Supplementary material for: The Kinetochore Protein Spc105, a Novel Interaction Partner of LaeA, Regulates Development and Secondary Metabolism in Aspergillus flavus
Source: Front Microbiol. 2019 Aug 13;10:1881. doi: 10.3389/fmicb.2019.01881 (PMC6700525; doi:10.3389/fmicb.2019.01881)
Supplement: TABLE S5 — Differentially expressed oxidative stress response genes in Δspc105 relative to WT. [file Table_5.DOCX]

| **Table S5 Differentially expressed oxidative stress response genes in *Δspc105* relative to WT (*p* < 0.01)** | | | |
| --- | --- | --- | --- |
| **Gene description** | **Gene locus** | **log2 fold change** | **Up/Down** |
| Superoxide dismutase | AFLA_099000 | 3.3895 | up |
| Mn superoxide dismutase MnSOD | AFLA_033420 | -6.1619 | down |
| Catalase Cat | AFLA_100250 | 2.9956 | up |
| Spore-specific catalase CatA | AFLA_056170 | 1.7823 | up |
| Glutathione oxidoreductase Glr1 | AFLA_083370 | 3.3988 | up |
| Glutathione peroxidase | AFLA_079910 | 1.9100 | up |
| Glutathione-S transferase GstA | AFLA_031820 | 2.6269 | up |
| Glutathione-S transferase theta, GST | AFLA_005530 | -4.2490 | down |
| Cytochrome c peroxidase Ccp1 | AFLA_110690 | 2.8538 | up |
